# Numerical study on the effect of capacitively coupled electrical stimulation on biological cells considering model uncertainties

**DOI:** 10.1038/s41598-022-08279-w

**Published:** 2022-03-18

**Authors:** Julius Zimmermann, Richard Altenkirch, Ursula van Rienen

**Affiliations:** 1 Institute of General Electrical Engineering, University of Rostock, 18051 Rostock, Germany; 2 Institute of Physics, University of Rostock, 18059 Rostock, Germany; 3 Department Life, Light and Matter, University of Rostock, 18051 Rostock, Germany; 4 Department of Ageing of Individuals and Society, Interdisciplinary Faculty, University of Rostock, 18051 Rostock, Germany

**Keywords:** Computational biophysics, Regenerative medicine, Tissue engineering, Computational models, Biomedical engineering, Biological physics

## Abstract

Electrical stimulation of biological samples such as tissues and cell cultures attracts growing attention due to its capability of enhancing cell activity, proliferation, and differentiation. Eventually, a profound knowledge of the underlying mechanisms paves the way for innovative therapeutic devices. Capacitive coupling is one option of delivering electric fields to biological samples that has advantages regarding biocompatibility. However, its biological mechanism of interaction is not well understood. Experimental findings could be related to voltage-gated channels, which are triggered by changes of the transmembrane potential. Numerical simulations by the finite element method provide a possibility to estimate the transmembrane potential. Since a full resolution of the cell membrane within a macroscopic model would lead to prohibitively expensive models, we suggest the adaptation of an approximate finite element method. Starting from a basic 2.5D model, the chosen method is validated and applied to realistic experimental situations. To understand the influence of the dielectric properties on the modelling outcome, uncertainty quantification techniques are employed. A frequency-dependent influence of the uncertain dielectric properties of the cell membrane on the modelling outcome is revealed. This may have practical implications for future experimental studies. Our methodology can be easily adapted for computational studies relying on experimental data.

## Introduction

**Figure 1:**
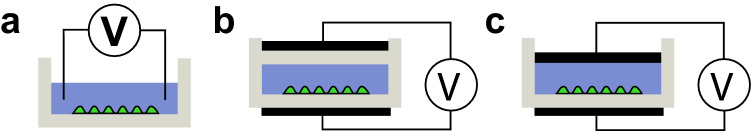
Overview of electrical stimulation approaches for in vitro cell culture experiments^10^. The Petri dish / insulator (grey) is shown together with the cell culture medium (blue), the cells (green) and the electrodes (black). (**a**) Direct contact stimulation, where the electrodes are in immediate contact with the cell culture medium. This may affect the sample, e.g. by altering the sample’s configuration as a result of chemical reactions at the electrode. Direct contact experiments are often performed using direct current (DC) signals^10^ or low-frequency waves of, for example, ${20}\,\hbox {Hz}$^13^. (**b**) Capacitive coupling remedies this drawback by isolating the electrodes from the sample. The electrodes can, for example, be placed outside a Petri dish that contains the sample^14,15^. Capacitive coupling requires higher frequencies (such as ${60}\,\hbox {kHz}$) to induce electric fields through the insulating material^16–18^. (**c**) Semi-capacitive coupling refers to a set-up where one electrode is in contact and the other one is isolated from the sample.

In the field of tissue engineering and regenerative medicine, researchers are always on a quest for new therapeutic approaches. One of these approaches is the electrical stimulation of biological samples and tissue^1–12^. A biological sample could be a piece of human-derived tissue or cells in a particular environment (culture medium or scaffold). The scope of the stimulation is varied. There are two main goals in tissue engineering: on the one hand, the differentiation of stem cells shall be guided by electrical stimulation. On the other hand, the regeneration of the extracellular matrix by enhanced protein expression of the stimulated cells is desired. To achieve these goals, three different experimental electrical stimulation approaches are usually considered (Fig. 1)^10,11^.

Among these three approaches, capacitive coupling has the highest biocompatibility since it effectively excludes electrochemical reactions at the electrode surface. It is used for the electrical stimulation of cartilage^14^, bone^19^, and their respective cells (osteoblasts^20^ and chondrocytes^7,15^).

Experimental studies have been conducted to evaluate the effect of the electric field on the cells. Two dominant pathways have been determined. They involve

1. voltage-gated channels^7,10,21,22^, which are triggered by a change of the transmembrane potential (TMP) of about ${100}\,\hbox {mV}$^10^. Other studies consider a change of the TMP by ${1}\,\hbox {mV}$ to be sufficient to induce a biological effect^23^. In a recent work, it has been speculated that a small change of a few mV of the TMP might not open the channels but ions would be driven into the cell due to the potential difference along the membrane^24^.
2. stretch-activated channels^10,25^, which may be activated by electroconformation or redistribution^25^.

By means of the finite element method (FEM), both the electric potential and electric field in electrical stimulation experiments can be numerically computed. In vitro experiments can be translated into numerical models by taking into account the geometrical dimensions as well as the material parameters^26^. Usually, the electric field on the macroscopic scale, for example, the field distribution in the Petri dish, is determined for electrical stimulation experiments. To make possible conclusions on the mechanism of interaction, the stimulated cells have to be considered as well. Due to the high aspect ratio between the cell membrane and the general setup, computations discretizing the detailed cell geometry have been mostly carried out for 2D models^18,27^. However, approximate methods can be used to avoid a discretization of the cell membrane^28,29^. By employing such methods, simulations of 3D in vitro set-ups including cells are possible^28^. The TMP is included in the FEM solution and can be easily extracted. The TMP, which is computed, corresponds to the induced TMP, which adds to the resting membrane potential.

We employ this approximate method to estimate the effect of capacitively coupled electric fields on biological cells. In contrast to direct-contact configurations, which have previously been studied with this method^28^, the cell in the capacitively coupled case is assumed to be in direct contact with an insulator covering the electrodes and not entirely surrounded by conductive cell culture medium^18,27^. Thus, we first compare the approximate model to a full-fidelity model (i.e., with a fully discretised membrane) to show that the approximate method can also be used to describe capacitive coupling. We also consider the uncertainty of the cell’s dielectric parameters, which has not been taken into account in previous studies of cells exposed to capacitively coupled electric fields^18,27^. For this purpose, we use efficient uncertainty quantification (UQ) techniques, which have the potential to be easily reused in future numerical studies on the effect of electric fields on biological cells. Furthermore, we show how experimental data and their uncertainty can be considered in numerical models to shed light on their reliability. Finally, we give an outlook on the usability of the presented approach for simulations of still simplistic, but realistic 3D configurations.

## Results

**Figure 2:**
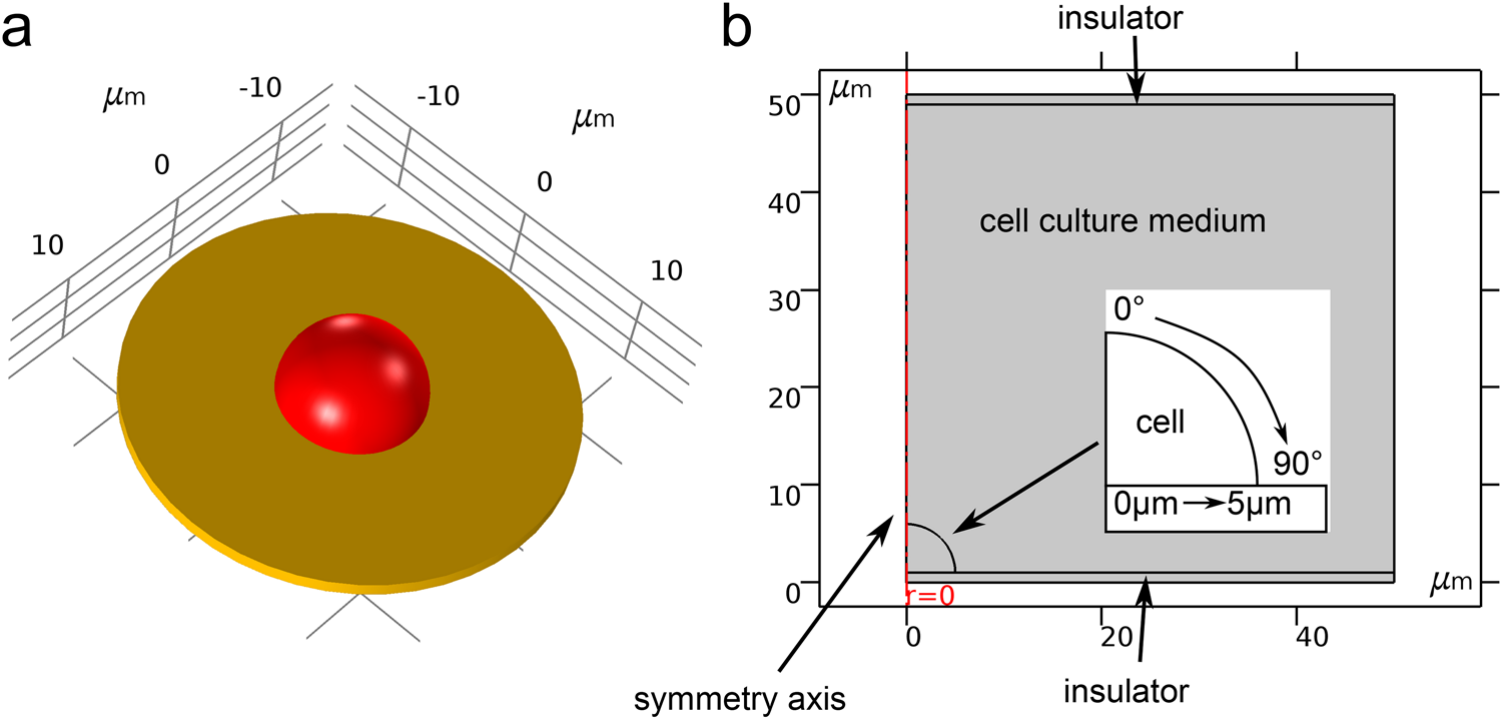
2.5D model of a cell on a substrate exposed to capacitively coupled fields^18^. The 3D equivalent of the 2.5D model (zoomed-in) is shown in (**a**). The cell (red) adheres to a substrate (yellow) which is a plastic insulator with a thickness of ${1}\,\upmu \hbox {m}$. The cell has a radius of ${5}\,\upmu \hbox {m}$ and its membrane a thickness of ${5}\,\hbox {nm}$. The 2D view of the 2.5D model is shown in (**b**). On the top and bottom boundaries of the domain, Dirichlet boundary conditions are applied to impose a net voltage difference. The boundary conditions mimic the electrodes, which are not explicitly modelled. Note that the electrodes are not in direct contact with the medium since they are covered by insulators. The other boundaries are electrically insulating. Material parameters for the cell cytoplasm and the culture medium are assigned as stated in Table 1. Different locations along the curved part of the cell membrane are denoted by the angle with the symmetry axis. Positions along the bottom part are denoted by the distance to the cell centre.

**Figure 3:**
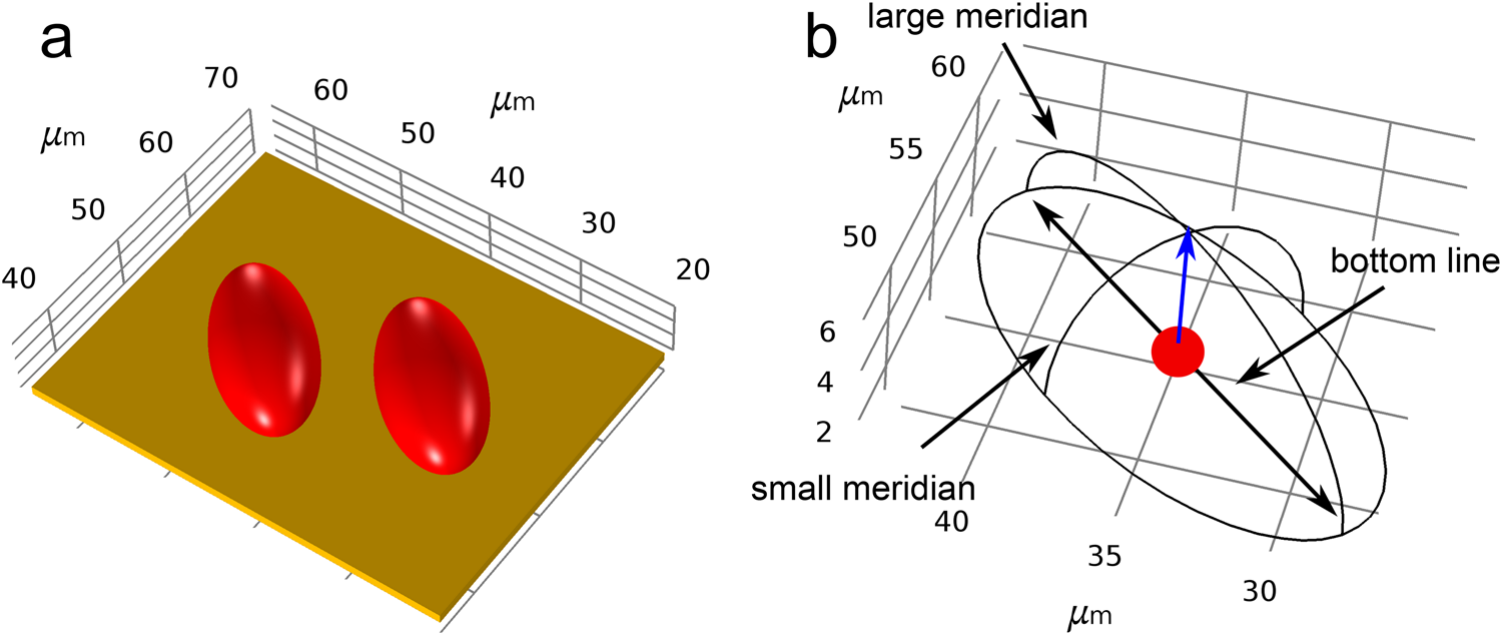
In 3D, cells with a semi-ellipsoidal shape were used to simulate the case of adherent cells. The model of two cells with a minimal distance of about ${5}\,\upmu \hbox {m}$ (zoomed-in) is shown in (**a**). The wireframe view of a single cell and the definition of lines along which the solution was evaluated are shown in (**b**). Points located on the small and large meridian were characterised by the angle between the reference vector (blue arrow) and the vector from the origin (red point) to the point on the membrane. The points along the bottom line were characterised by the distance to the centre point.

In this work, we consider two geometrical models: (a) a hemispherical cell (Fig. 2) and (b) two semi-ellipsoidal cells (Fig. 3) situated on the bottom of a cell culture well. The hemispherical cell is an abstract model, which has been considered in previous works^18^. The semi-ellipsoidal cell shape has been shown to approximately represent adherent cells^28^. Both geometries correspond to a capacitive-coupling set up as shown in Fig. 1c. Due to the symmetry of the hemispherical cell geometry, it can be described by a 2D geometry with corresponding symmetry boundary conditions. In the following, we refer to this model as 2.5D model (more details are given in the “Methods” section). The two semi-ellipsoidal cells cannot be described by a 2.5D model and are thus modelled in 3D. In previous studies, the cell membrane has been discretised explicitly^18,23,27^. Such models are referred to as full-fidelity models since they do not make any approximations. In addition to the full-fidelity model, we also consider an approximate method that takes the cell membrane implicitly into account^28^. In this study, the conductivity $\sigma ({\mathbf {r}}, \omega )$ and permittivity $\varepsilon ({\mathbf {r}}, \omega )$ were assumed to be constant in the respective subdomains of the model. Moreover, the frequency dependence of the individual dielectric properties was neglected, which for materials similar to the ones considered here is a valid assumption for frequencies of up to about 100 MHz (Fig. S1). The frequency range, in which the assumptions of the electro-quasistatic regime are valid, is limited by the dielectric properties and characteristic length of the system under investigation^30,31^. For the systems studied by us, an electromagnetic wave propagation violating these assumptions made is certainly not to be expected for frequencies below ${100}\,\hbox {MHz}$ (Fig. S2). In tissue-engineering experiments employing capacitive coupling, frequencies in the $\hbox {kHz}$ range are used, for example, for a stimulation of cartilage cell cultures^7,15,32^ or cartilage explants^14,33,34^. Hence, we considered frequencies ranging from ${10}\,\hbox {Hz}$ to ${100}\,\hbox {MHz}$ to also include frequencies that have not been considered in experiments yet.

### Validation of the numerical model and studying the influence of the membrane conductivity

Before applying UQ techniques and allowing for experimentally determined material parameters, we focus on the validation of the numerical approach. We performed an analytical estimation of the electric field in the cell culture medium. The deviation between the analytical and the numerical solution is negligibly small (Fig. S7). However, the relative error grows with decreasing frequency below ${100}\,\hbox {Hz}$.

To check the applicability of the approximate method for the cell model, the results generated using the approximate method were compared against the results obtained by the so-far employed full-fidelity model at prominent points along the cell membrane. The TMP in the electro-quasistatic formulation is a phasor. Thus, its absolute value and phase were computed and compared. Apart from this comparison, we generally report the absolute value of the TMP as this is the property of interest in therapeutic applications.

Firstly, the TMP was computed for the same dielectric parameters as in previous studies^18,27^ (see Table 1) using the full-fidelity as well as the approximate model. The approximate and the full-fidelity method did not deviate significantly (see also Figs. S8–S15). Furthermore, we observed that the accuracy of the approximate method does not deteriorate when a conductivity greater than ${0}\,\hbox {S}/\hbox {m}$ was chosen. Hence, we concluded that the approximate method works reliably in a capacitive-coupling setting when the cell is not only surrounded by conductive cell culture medium but also in close contact with an insulator. Consequently, we only report the results of the approximate method in the following.

**Figure 4:**
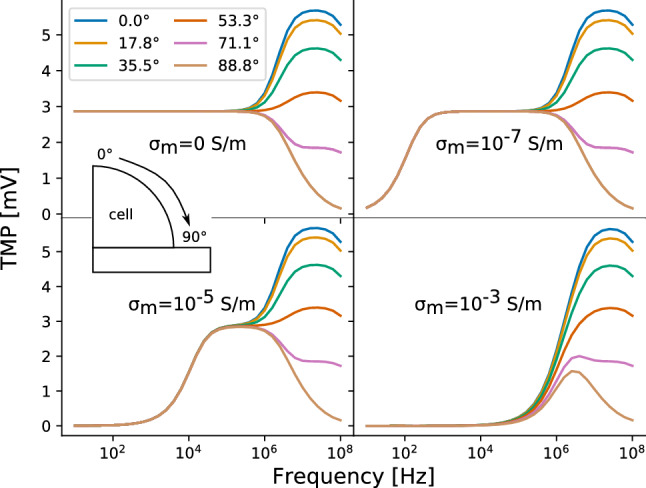
TMP along the curved part of the cell membrane for different membrane conductivities of ${0}\,\hbox {S}/\hbox {m}$, ${10^{-7}}\,\hbox {S}/\hbox {m}$, ${10^{-5}}\,\hbox {S}/\hbox {m}$ and ${10^{-3}}\,\hbox {S}/\hbox {m}$. The results were generated using the approximate method.

In previous studies^18,27^ the idealised case of a membrane conductivity of ${0}\,\hbox {S}/\hbox {m}$ was assumed. Realistic values of the membrane conductivity are in the range of ${10^{-5}}\,\hbox {S}/\hbox {m}$ to ${10^{-8}}\,\hbox {S}/\hbox {m}$ as the membrane is not a perfect insulator and permits a leakage current^35–37^. It turns out that changes in the membrane conductivity strongly influence the results (Fig. 4). Upon alteration of the membrane conductivity, a high-pass-filter effect was observed. In the idealised case of ${0}\,\hbox {S}/\hbox {m}$, the TMP is constant and non-zero over a broad frequency range. This would indicate an effective stimulation already at very low frequencies approaching the static limit of 0 Hz. Our results using a non-zero membrane conductivity suggest, that the TMP is close to zero for low frequencies before it starts to approach the constant value that was predicted previously. Nevertheless, this constant value, which is usually approached between 1 kHz and 1 MHz, is independent of the conductivity (if the TMP rises at a sufficiently low frequency). The greater the membrane conductivity, the higher becomes the frequency, from which on the TMP rises. In the high-frequency limit, the values again coincide irrespective of the membrane conductivity. Later, we will show that the TMP in this frequency region is mainly influenced by the membrane permittivity, which explains the aforementioned observation.

The absolute value of the TMP depends on the location on the membrane for frequencies above about ${1}\,\hbox {MHz}$. Figure 4 shows that at the membrane apex (denoted by the blue line, i.e. an angle of ${0}^{\circ }$), the TMP increases from about ${1}\,\hbox {MHz}$ and peaks at about ${10}\,\hbox {MHz}$ before it decreases. A special point on the membrane is the triple point, where membrane, medium and insulator meet (see also Fig. S4). This point is a result of the geometrical modeling, in which the cell builds a relatively sharp corner with the insulator and the medium. Such a corner is most likely not present in reality. On the circular part, the triple point is located at an angle of ${90}^{\circ }$. Close to this point, the TMP drops continuously from about ${1}\,\hbox {MHz}$ on and does not peak. These results are mostly caused due to the geometrical modeling, so this behaviour probably has numerical reasons. The TMP at these points should not be considered representative of the electrical stimulation.

**Figure 5:**
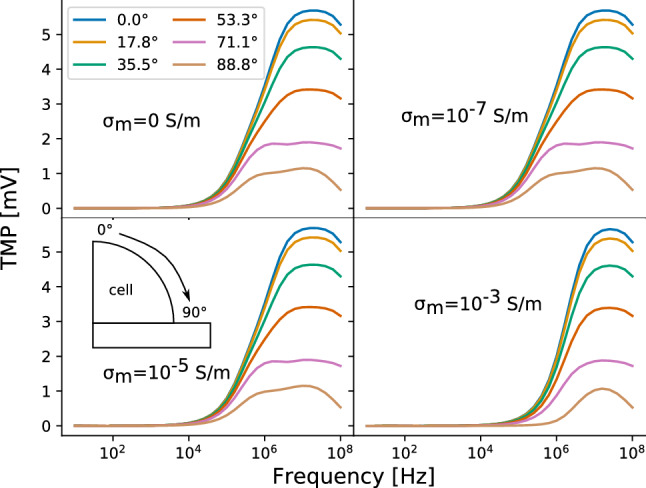
TMP along the curved part of the cell membrane for different membrane conductivities of ${0}\,\hbox {S}/\hbox {m}$, ${10^{-7}}\,\hbox {S}/\hbox {m}$, ${10^{-5}}\,\hbox {S}/\hbox {m}$ and ${10^{-3}}\,\hbox {S}/\hbox {m}$ when the cell was separated from the well bottom by a gap of 100 nm. The results were generated using the approximate method.

When the TMP along the bottom line is considered, qualitatively similar observations could be made (Figs. S16 and S17). The higher the membrane conductivity is, the higher frequencies are needed to induce a non-zero TMP. The TMP along the bottom is roughly 1.9 times larger than that along the circular part. It does not depend much on the location on the cell membrane except for points close the triple point.

In recent years, the numerical simulation models of capacitive-coupling stimulation have assumed that cells are in direct contact with the insulator on the electrode^18,27^. However, it has been found in experiments that there exists a gap between the cell membrane and the substrate. For neurons, the gap is homogeneous, i.e. has an almost constant thickness between 60 nm and about 100 nm^38^. The gap is most likely filled with a conductive electrolyte with at least the same conductivity as the cell culture medium^39^. We are not aware of a study investigating the adhesion of, for example, chondrocytes or osteoblasts at such a fine scale. Thus, we included a gap of 100 nm filled with cell culture medium in the aforementioned 2.5D model. The results of this model are shown in Fig. 5 and can be directly compared to the results shown in Fig. 4 (i.e., the case without a gap). The shape of the TMP curves of the model without (Fig. 4) and with the gap (Fig. 5) are very similar at very high frequencies greater than about 1 MHz for most of the points on the membrane. The TMP is mostly zero at lower frequencies for all membrane conductivities if the gap is considered. The frequency from which on the TMP becomes non-zero only slightly increases with increasing membrane conductivity. Evidently, the gap has a similar effect as the highest membrane conductivity in the model without a gap. In the following, we will not further consider the gap to keep our modelling approach comparable to previous studies^18,27^ and due to a lack of reliable experimental data to be used in an UQ approach.

### Uncertainty quantification

#### Preliminary uncertainty quantification of a reduced model

UQ permits to analyse the influence of more than one parameter on the modelling outcome. Initially, we conducted a UQ analysis using probability distributions for the parameters based on our a priori knowledge (see Table 2). The Python toolbox *Uncertainpy*^40^ was used to generate a set of input parameter combinations according to the assumed probability distributions. A surrogate model is then generated from the individual modelling results and yields statistical measures to characterise the modelling outcome with respect to the uncertainty in the input parameters.

**Figure 6:**
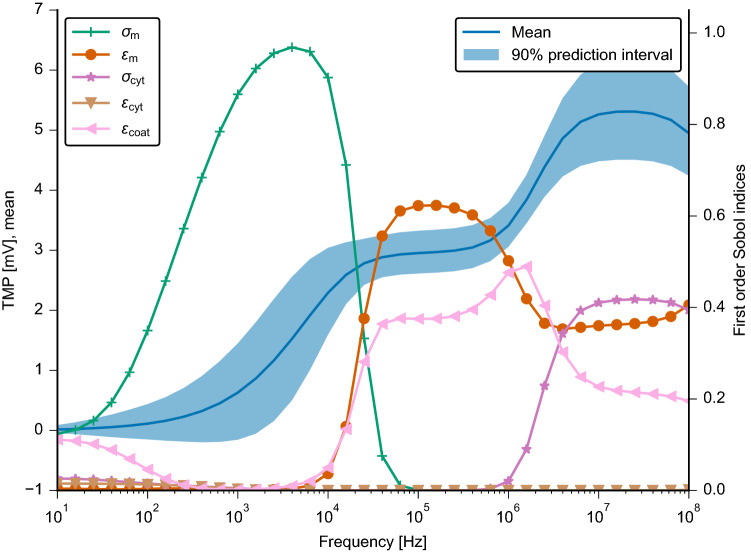
Left axis: Mean and $90\%$ prediction interval of the absolute value of the TMP at the cell apex for the basic model shown in Fig. 2. Right axis: First order Sobol indices for each uncertain parameter, i.e. the conductivity (dark green) and permittivity (orange) of the membrane, the conductivity (purple) and permittivity (brown) of the cytoplasm, and the permittivity of the coating (pink), respectively.

The result of the UQ analysis for the simple cell model (Fig. 2) is shown in Fig. 6. Here, the focus was set on the TMP value at the cell apex, i.e. the highest point of the cell where the TMP becomes maximal on the circular part of the membrane. A measure for the sensitivity of the modelling outcome with respect to individual input parameters are the so-called Sobol indices^40^ (see a more detailed explanation in the “Methods” section). We did not observe a significant difference between first-order and total Sobol indices in regions where the TMP was considerably larger than zero. This indicates no significant interaction between the input parameters. Consequently, we only report first-order indices in the following.

The mean value of the TMP behaves similarly, as previously shown in Fig. 4 for non-zero membrane conductivity. Its $90\%$ prediction interval is not broader than ${1}\,\hbox {mV}$ for all frequencies. It highlights the possible range in which the TMP value can be expected. Thanks to the first-order Sobol indices, the possible deviation from the mean TMP can be attributed to the different parameters. In the range up to about ${10}\,\hbox {kHz}$, the membrane conductivity $\sigma _{\mathrm {m}}$ plays a crucial role. From ${10}\,\hbox {kHz}$ to ${1}\,\hbox {MHz}$, the TMP is most sensitive to changes of the membrane permittivity $\varepsilon _{\mathrm {m}}$. In addition, the permittivity of the coating $\varepsilon _{{\mathrm {coat}}}$ plays a significant role. For higher frequencies, membrane permittivity, coating permittivity, and cytoplasm conductivity $\sigma _{{\mathrm {cyt}}}$ contribute to a change of the TMP with their uncertainty. Furthermore, the cytoplasm permittivity $\varepsilon _{{\mathrm {cyt}}}$ does not have any influence over the entire frequency range. We also investigated the TMP at the centre point at the bottom of the cell, i.e. where the cell is in direct contact with the insulator (Fig. S18). The main difference to the result at the cell apex is that the cytoplasm conductivity does not influence the TMP at higher frequencies. As mentioned previously, the TMP is greater at the cell bottom and does not further increase at frequencies above ${1}\,\hbox {MHz}$. Since changes of the cytoplasm permittivity did not reveal any influence on the modelling outcome, we did not further consider it in our analysis.

In the previous paragraph, assumptions were made on the probability distributions of the uncertain parameters. The range of the parameters was chosen to include the values of previous numerical studies^18,27^. A more realistic result of the UQ was gained by using probability distributions based on experimental data and their reported uncertainties^37^ (Table 3). This study focused on chondrocytes because they have been subjected to capacitively coupled fields^14,15,34^ to develop cartilage tissue engineering approaches. The choice of chondrocytes is also supported from a clinical perspective. Cartilage regeneration poses an open challenge while bone regeneration is better understood^41^. While capacitive-coupling stimulators are used to treat, for example, non-union of bones^12^, no such clinical application of capacitively coupled fields for cartilage regeneration exists despite promising in vitro results^14,15,34^. The experimentally determined parameters for chondrocytes differ in two aspects from the previously chosen parameters: their probability distributions are normal distributions instead of uniform distributions and the expectation values for the cytoplasm conductivity as well as membrane permittivity are outside the previously tested interval. At this point, we would like to mention that the cytoplasm conductivity chosen in previous numerical studies of capacitive coupling stimulation^18,27^ (and thus used by us in the previous paragraph) is relatively large compared to the expected values for eukaryotic cells^36^. In contrast, the experimentally determined values^37^ are in the expected range.

**Figure 7:**
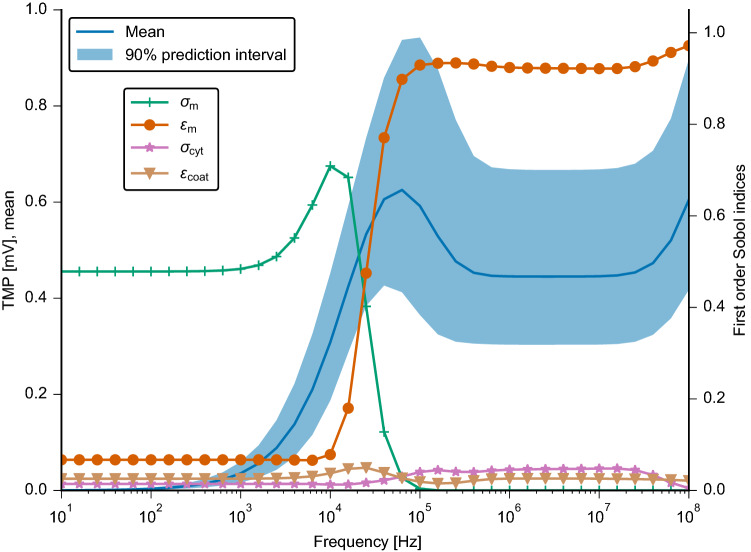
Left axis: Mean and $90\%$ prediction interval of the absolute value of the TMP at the cell apex for the basic model shown in Fig. 2. Right axis: First order Sobol indices for each uncertain parameter, i.e. the conductivity (dark green) and permittivity (orange) of the membrane, the conductivity (pink) of the cytoplasm, and the permittivity of the coating (brown), respectively.

The results using the dielectric properties of chondrocytes are shown in Fig. 7. It is evident that the TMP is considerably smaller when using the experimentally determined parameter values. Moreover, the shape of the 90% prediction interval and the course of the mean value of the frequency-dependent TMP differ from our previous results using guessed properties (Fig. 6). The mean value peaks between ${30}\,\hbox {kHz}$ and ${90}\,\hbox {kHz}$. At the same frequencies, the prediction interval covers the highest possible TMP values. Close to the maximal frequency of ${100}\,\hbox {MHz}$, the TMP increases again. Below ${10}\,\hbox {kHz}$, the TMP value tends to zero with high probability.

As presented in the previous paragraph, the membrane conductivity plays a dominant role in the low-frequency range, whereas the membrane permittivity influences the results most in the high-frequency range. Interestingly, the comparatively small error of the cytoplasm conductivity also leads to a decreased influence of this parameter. The same holds true for the coating permittivity. The result at the cell bottom (Fig. S19) resembles the result at the cell apex. A significant difference is that the TMP does not peak, but instead remains almost constant from about ${60}\,\hbox {kHz}$ onwards. Furthermore, the TMP value is larger and the influence of the cytoplasm conductivity is even smaller.

#### Uncertainty quantification of a realistic 3D model

To validate the possibility and physical correctness of the modelling of a 3D geometry using the approximate method, we compared the 2.5D model shown in Fig. 2 to its 3D representation. The comparison showed good agreement between the two models with deviations of a few percent or less or a very small absolute error. The results deviated notably only close to the triple point.

As a more realistic model of adherent cells, we considered the model shown in Fig. 3. Since an evaluation of the model over the same frequency range as in the previous section becomes prohibitively expensive even on well-equipped workstations, we focussed on one frequency. We chose ${60}\,\hbox {kHz}$, since this frequency has often been used in experiments with chondrocytes so far^14,15,34^. We performed UQ on this model using the experimentally determined parameters for chondrocytes (Table 3). Based on the results of the reduced model (Fig. 7), we omitted the cytoplasm conductivity and the coating permittivity to further reduce the complexity of the UQ analysis.

**Figure 8:**
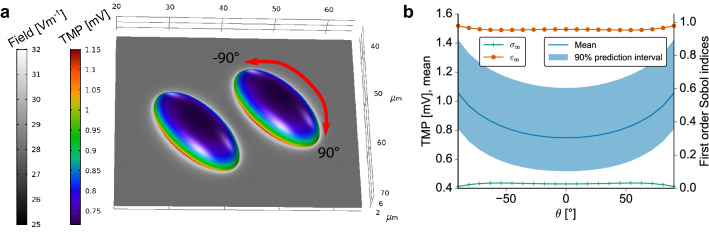
(**a**) The electric field strength around the cells and the TMP on the cell surface are shown for the expectation values of the cell dielectric properties (Table 2). (**b**) Left axis: Mean and $90\%$ prediction interval of the absolute value of the TMP at the cell apex for the ellipsoid cell model shown in Fig. 3. Right axis: First order Sobol indices for each uncertain parameter, i.e. the conductivity (dark green) and permittivity (orange) of the membrane. The results were evaluated along the large meridian using the angle with the central normal vector as an indicator for the location [as indicated by the red arrow in (**a**)]. The results along the small meridian deviated only slightly and are not reported.

**Figure 9:**
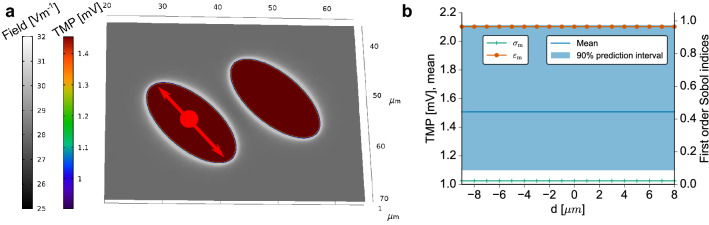
(**a**) The electric field strength around the cells and the TMP on the cell bottom are shown for the expectation values of the cell dielectric properties (Table 2). (**b**) Left axis: Mean and $90\%$ prediction interval of the absolute value of the TMP at the cell bottom for the ellipsoid cell model shown in Fig. 3. Right axis: First order Sobol indices for each uncertain parameter, i.e. the conductivity (dark green) and permittivity (orange) of the membrane. The results were evaluated along the bottom line of the cell using the distance to the centre as an indicator for the location [as indicated by the red arrow in (**a**)].

To better understand the stimulation of the two cells, the electric field strength around the cells and the TMP on the cell surface are considered (Fig. 8a). The observables were computed for the expectation values of the model parameters. It turns out that the field is mostly homogeneous around the cells and changes only in close proximity to the individual cells. It is in agreement with this observation that the TMP appears to be identical on both cells. Consequently, we only report the results for one cell since we observed no significant numerical deviation between the results for each of the two cells. In comparison to the results at ${60}\,\hbox {kHz}$ using the previously assumed dielectric properties (Fig. 4), the TMP changes on the curved part of the cell surface (Fig. 8a). It increases with decreased distance to the cell bottom, i.e. it does not assume its maximal value at the cell apex. Instead, the TMP close to the triple point is about $25\%$ greater than at the cell apex. However, on the cell bottom it remains constant (Fig. 9a), which was also the case in our previous analysis of the reduced model. We could attribute the change in the spatial dependency of the TMP on the curved part of the membrane solely to the change of the chosen dielectric properties. The validity and accuracy of the approximate method was not influenced when choosing the chondrocyte dielectric properties instead of the previously assumed properties.

When analysing the influence of the modelling outcome in a UQ setting, deviations of the TMP with respect to the membrane properties could be observed (Figs. 8b and 9b). Again, changes of the membrane permittivity influence the TMP strongly while the membrane conductivity plays a subordinate role, given the uncertainty of the experimental data. The 90% prediction interval of the TMP is rather wide and can extend to values that are 40% larger than the mean TMP. Since we used homogeneous, i.e. spatially independent, dielectric properties on the cell membrane, the width of the prediction interval does not depend on the specific location on the cell membrane. However, the aforementioned spatial dependency along the curved part of the membrane is reflected in both the mean TMP and the prediction interval (Fig. 8b). Accordingly, the mean TMP and prediction interval along the cell bottom are not spatially dependent (Fig. 9b).

## Discussion

The application of capacitively coupled electric fields in the context of tissue engineering has been experimentally demonstrated to promote, for example, osteogenesis^3,8,42^ or chondrogenesis^5,8,10,12,15^. However, the exact way how cells react to external electric fields is not known. In this paper we focus on the theoretical description of a capacitively coupled stimulation system to advance the knowledge of numerical simulations of such systems. The numerical results of the studied benchmark model are in good agreement with previously published results^18^. Note that the electric field across the membrane (which other publications^18,27^ focussed on) can be calculated by dividing the TMP by the membrane thickness. Likewise, the TMP of about ${3}\,\hbox {mV}$ for a hemispherical cell with idealised membrane conductivity corresponds to an electric field strength of ${0.6}\,\hbox {MV}/\hbox {m}$ for a membrane of ${5}\,\hbox {nm}$ thickness. This result of the 2.5D simulation is about ${0.2}\,\hbox {MV}/\hbox {m}$ less than reported for the pure 2D case^18^. Hence, it shows how important the axisymmetric assumption is to account for the real 3D geometry.

Furthermore, we considered an approximate method to avoid the explicit meshing of the cell membrane (full-fidelity approach). The approximate method allows us to keep the accuracy of the numerical model while significantly increasing the computational efficiency. It is a reliable alternative to the full-fidelity membrane model. What is more, it even paves the way for simulations of realistic 3D geometries. A direct comparison of the two methods revealed that only the phases deviated slightly in the low-frequency range (see also Supplementary Information). This could be due to a numerical stability problem of the employed direct solver for the full-fidelity approach. The ratio between the small elements of the discretised membrane and the larger elements in the rest of the domain makes the system hard to solve for low frequencies^43^. In COMSOL Multiphysics®, this is indicated by a refinement warning. This warning was not raised with the approximate method, which in this sense appears to be numerically more stable. The exact implementation and thus accuracy of the underlying numerical method is not known. Theoretical considerations on the implementation of such an approximate method in a FEM framework can be found elsewhere^44–46^. The use of second-order Lagrange finite elements in COMSOL Multiphysics® suggests that a third-order accurate computation of the TMP can be expected^45,46^. Thus, we would expect the approximate method to be theoretically more accurate than finite differences approaches^47^ or other FEM approaches^48,49^. To identify how accurate the considered FEM approach can represent the experimental situation, experimental validation is required. The validation by comparison to an equivalent circuit provides the opportunity to bridge the gap between experiment and theory in the future. Impedance spectroscopy is a tool that permits the determination of the linear electric properties of the entire system. The measured data can then be fitted to an equivalent circuit model or can be compared to the numerically determined impedance^50^.

The approximate method reduces the computational cost so that a sensitivity analysis of the model by UQ techniques becomes easily feasible. UQ techniques require a frequent repetition of the simulation run to sample the parameter space. Nevertheless, the total runtime of the UQ study with the approximate model was eventually of the same order as the runtime of the full-fidelity model.

The UQ analysis is motivated by our finding that the model outcome strongly depends on the membrane conductivity. Speaking in terms of a filter, it seems that there exists a cut-off frequency that depends on the membrane conductivity. UQ offers a possibility to study the influence of several parameters at once. This improves on a previous approach^18^, where the influence of one parameter at a time but not the combined influence of many input parameters has been studied.

By studying a simple, reduced system such as the 2.5D model of a hemispherical cell, the influence of different parameters can be studied. Eventually, individual parameters can be omitted in UQ analyses of more complicated and computationally expensive models if they do not reveal a significant influence on the reduced, cheaper model. However, this choice cannot be generalised but must be made dependent on the known or assumed parameter uncertainties. As we showed, in the case of assumed parameter uncertainties the cytoplasm conductivity and coating permittivity had a significant influence in certain frequency regions. In contrast, both parameters had no influence in the case of uncertainties inferred from experimental parameters. Thus, the choice of which particular parameters to omit in the analysis of a more sophisticated model is not trivial.

The magnitude of the TMP changed significantly when the assumptions on the material properties were altered. In any case, it was in the $\hbox {mV}$ range that is assumed to possibly have a biological effect^23,24^. In the case of chondrocytes and related stem cells, the biological effect corresponds to a remedy of de-differentiation^5,15^ and enhanced chondrogenic differentiation^10^. It is to be investigated if the induced TMP is sufficiently large to trigger voltage-gated channels in chondrocytes, which have been identified as crucial for signal transduction in chondrocytes stimulated by capacitively coupled fields^7^. Ideally, reliable TMP thresholds can be determined such that validated numerical simulations can be used to tailor the stimulation regarding the experimental reality (i.e., geometry, bio/electro/chemical environment).

Similar conclusions can be drawn from both UQ models, which differ in the assumed probability distributions. In the case of a leaky membrane, i.e. a membrane that permits a leakage current due to a non-zero conductivity, there exists a cut-off frequency below which the TMP tends to zero. Above this frequency, the TMP assumes a value that could be physiologically relevant. Thus, we speculate that the electrical stimulation should not be effective below the cut-off frequency. Indeed, finding a suitable stimulation protocol is a frequent research objective^5,15,20^. Our approach could facilitate the choice of the right frequency. We found that for a cell membrane conductivity of down to ${10^{-7}}\,\hbox {S}/\hbox {m}$, the stimulation frequency should be above ${1}\,\hbox {kHz}$ (Fig. 4). For chondrocytes, which are a target of capacitively coupled electrical stimulation^5,14,15,32^, a frequency above ${10}\,\hbox {kHz}$ but not exceeding ${100}\,\hbox {kHz}$ could be most efficient (Fig. 7). This conclusion is in good agreement with the experimental findings^5^ that led to the establishment of ${60}\,\hbox {kHz}$ as the stimulation frequency in the capacitively coupled stimulation of chondrocytes^15^. Furthermore, a decrease of the TMP with increased membrane conductivity could be found experimentally for DC stimulation^51^. The membrane permittivity does not contribute to the cut-off effect. It only leads to a change of the TMP value. The aforementioned cell models rely on the assumption that the cell membrane is in direct contact with the insulator. Introducing a small gap between the cell and the electrode had a similar effect as increasing the membrane conductivity. Hence, we speculate that an adhesion gap between cells and the insulator, which is filled with a conductive electrolyte, also leads to a cut-off effect. Due to limited availability of experimental data, we did not further investigate the model with the gap and the influence of its geometrical and dielectric properties on the model result. For future experimental validation approaches, it should be kept in mind that the gap might play a significant role and thus should be carefully quantified.

As a realistic 3D model, we chose a model of two hemiellipsoids in close proximity motivated by previous findings that hemiellipsoids approximately represent adherent cells^28^. Since we identified the conductivity and permittivity of the cell membrane as the key parameters for estimating the TMP, we only analysed the 3D model with respect to these parameters. Furthermore, we only chose the frequency that has often been chosen in experiments. These two simplifications reduce the complexity of the UQ analysis considerably and make it possible to tackle such a complex model. Since we found no difference between the two cells, they evidently do not seem to influence each other during the stimulation. This model describes the case of rather freshly seeded cells that have not yet grown into a monolayer. For such cases, different models have to be built based on imaging data. Subsequent tests will then demonstrate whether our observation also holds true for different distances between the cells and multiple, irregularly shaped cells interacting with each other.

We found no preference of a meridian and in general no strong dependence of the TMP on the location on the cell membrane (Figs. 8 and 9). Unlike in the case of spherical cells in an external field^28^, this TMP does not have poles where it becomes zero. We assume that this is due to the difference in stimulation and the direct contact of the cells with the insulating coating layer. For all configurations studied in this paper, the TMP was greater along the cell bottom than at the top side of the cell. Without experimental evidence it remains unclear whether this can be related to the biological effect of the electrical stimulation. We found that the relative permittivity of the cell membrane strongly influences the TMP at the relevant frequency of ${60}\,\hbox {kHz}$. A comparison of different cell types with different membrane permittivities would be an interesting subject for further research in order to demonstrate whether the stimulation effect can be related to this parameter.

The induced TMP can be, for example, measured by fluorescence microscopy with the help of potentiometric dyes. For direct contact stimulation with much larger field strengths as used in capacitive coupling, numerical simulations could be validated by fluorescence spectroscopy experiments^28^. Moreover, this experimental approach permits to assess the spatial dependency of the TMP. Our numerical results suggest that the spatial dependency of the TMP depends on the dielectric properties of the cell membrane and cell cytoplasm. Furthermore, measurements of the TMP at low frequencies in the Hz range could answer if the cut-off effect exists. Based on the results of our simulations, which account for a non-zero membrane conductivity, no significant TMP should be induced at these frequencies due to the external stimulation. In contrast, previous studies^18,27^ have predicted an induced TMP even for frequencies approaching 0 Hz. Thus, the measurement results could also be used to infer dielectric properties or detect changes in the physiological state of the cells, which manifest itself in changes of the dielectric properties. Besides the small field strengths and thus small TMP values, the high frequencies of the capacitively coupled fields in the kHz range pose a challenge for such optical validation approaches. An alternative experimental approach could be local impedance measurement. Recently, impedance measurements of single cells at 2 Hz using optical methods have been demonstrated^52^. The experimental results could qualitatively be explained by numerical simulations. With progress in this field, experimental validation of our models also at higher frequencies could become feasible. Furthermore, optical measurements could clarify if there is a mechanical deformation or interaction between the cells. The relevant properties to estimate the (mutual) forces on the cells can be readily extracted from FEM simulations^53,54^. Hence, the sensitivities of, for example, the force on the membrane with respect to the model parameters could also easily be computed using the presented UQ approach.

A limitation of the approximate method is the simplification of the cell membrane. In our model, the cell membrane is a perfectly smooth thin layer with a fixed conductivity. At high frequencies in the upper MHz-range, the dielectric properties of the membrane can become frequency-dependent^55^. We did not account for this relaxation, which has a characteristic relaxation frequency of about 180 MHz. The dielectric properties of chondrocytes have not been measured at such high frequencies to the best of our knowledge. In future works, the membrane relaxation can be straightforwardly integrated into the FEM approach by using frequency-dependent membrane conductivity and permittivity described by a relaxation model (e.g., the Debye model^55^). For the UQ analysis, the uncertainty of the parameters of the relaxation model can be considered. A nonlinear, TMP-dependent description of the membrane conductivity is common to model electroporation^56^. Then, the conductivity would become time-dependent. We do not expect electroporation during capacitively coupled stimulation. Thus, we did not use a nonlinear conductivity. In principle, the structure of (3) permits to assign position-dependent conductivity, permittivity or thickness values on the membrane. This could be exploited to account for stochastic fluctuations of the dielectric and geometric properties of the membrane. Alternatively, models using a rough membrane could be considered. Such models could, for example, contain membrane protrusions^57^ or an oscillating membrane thickness^58^.

Simplistic computational cell models such as the models considered here are commonly used across different communities^18,27,28,47,49,54,59^. More sophisticated models including a large number of cells require large high-performance-computing facilities^60^. For types of tissue such as cartilage, where the volume fraction of the cells is small and the distance between the cells is rather large^61,62^, models including few or only one cell might be sufficient. Nevertheless, in future studies, we intend to use 3D models for the numerical cell models that are extracted from in vitro experiments via computer-graphics based methods.

The presented UQ approach takes into account the assumed probability distributions of the material parameters and propagates them through the model. If reliable experimental data was available, the probability distribution of the model parameters could be inferred using a Bayesian approach, potentially exploiting the efficiency of PC methods^63^. This corresponds to solving the inverse problem. The presented FEM model could be reused for such an approach.

## Conclusion

Electrical stimulation is a promising therapeutic tool in regenerative medicine. In in vitro experiments, the reaction of individual cell cultures to external electric fields can be studied before translating the gained knowledge to the tissue level. However, experimental approaches are, to date, mostly dominated by trial-and-error. Here, we presented numerical models to shed light on the underlying mechanisms of interaction. We focussed on capacitive coupling as it has several benefits. By introducing an alternative approximate model for an already established simulation model, we were able to reduce the computational cost significantly without compromising the accuracy. Subsequently, we performed an efficient sensitivity study regarding dielectric cell properties and their individual influence. Our results reveal that the dielectric properties have a great influence and should be known as precise as possible. With accurate knowledge of the modelling parameters, our modelling approach can enable enhanced experimental designs.

In future research, we will advance efficient UQ techniques that supply fast UQ studies for 3D geometries. Within this framework we will transfer knowledge from our previous UQ study on a human brain model^64^ to the field of in vitro electrical stimulation. This UQ approach will enable other researchers to reuse our solution that is based on the open-source tool *Uncertainpy*. A possible application could be in the *TTField* community^59^ or in the electroporation community^29,47,48,56^, where similar cell models are used. Following the research presented in^44–46,65^, we will realise an open-source solution for the FEM model.

The application of theoretical multiphysics models requires the performance of experimental studies on the mechanisms of interaction. A great contribution would be a clarification of the question whether voltage-gated^7^ or other channels are involved in the signal transduction. This could lead to multiphysics models including, for example, ion dynamics^66^ or the mechanical behavior of the membrane^53,67^. Furthermore, the adhesion of chondrocytes and resulting gaps between cell membrane and insulator should be investigated to provide reliable geometric models of cells in capacitive-coupling stimulation chambers.

## Methods

### Computational electromagnetics

The electric field can be computed by solving Maxwell’s equations. Maxwell’s equations comprise a set of time-dependent, coupled partial differential equations, which fully describe magnetic and electric fields and their interaction. In many therapeutic approaches for biological systems, slowly varying electromagnetic fields can be assumed^31^. In the so-called electro-quasistatic regime^68^, the electric fields are curl-free and often time-harmonic. Thus, the magnetic fields and the eddy currents are negligible; the displacement current prevails. In this regime, the electric potential $\Phi$ for capacitive coupling with time-harmonic input signals can be described by the field equation

1 $$\begin{aligned} \nabla \cdot \left[ \sigma ^*({\mathbf {r}}, \omega ) \; \nabla \Phi ({\mathbf {r}}) \right] = 0 , \end{aligned}$$

where the complex conductivity $\sigma ^*$ equals $\sigma ({\mathbf {r}}, \omega ) + j \, \omega \varepsilon ({\mathbf {r}}, \omega )$. From the solution of (1), which can, for example, be obtained by FEM, the electric field can be computed as ${\mathbf {E}} = - \nabla \Phi$. For the processes on the cellular level, the TMP is the quantity of interest. It is defined as

2 $$\begin{aligned} {{\mathrm {TMP}}} = |\Phi _{\mathrm {o}} - \Phi _{{\mathrm {i}}}| , \end{aligned}$$

where $\Phi _{{\mathrm {i}}}$ is the potential inside the membrane and $\Phi _{{\mathrm {o}}}$ the potential outside the membrane.

In the conventional FEM approach, the entire geometry is discretised into geometrical elements, for example tetrahedra^69^. To avoid discretising the membrane with its small thickness $d_{{\mathrm {m}}}$, it can be represented by an inner boundary line that fulfils the condition

3 $$\begin{aligned} {{\mathbf {n}}} \cdot {{\mathbf {J}}}_{{\mathrm {o,i}}} = \pm \frac{\sigma ^*_{{\mathrm {m}}}}{d_{{\mathrm {m}}}} \left( \Phi _{{\mathrm {o}}} - \Phi _{{\mathrm {i}}} \right) = \mp \frac{\sigma ^*_{{\mathrm {m}}}}{d_{{\mathrm {m}}}} {{\mathrm {TMP}}} \end{aligned}$$

for the electric current density ${{\mathbf {J}}}_{{{\mathrm {o,i}}}}$ outside and inside the membrane, respectively^28^. Here, the normal vector on the membrane is denoted by ${{\mathbf {n}}}$. The complex conductivity of the membrane $\sigma ^*_{{\mathrm {m}}}$ contains its dielectric properties. As stated in (3), the TMP is already part of this formulation and can be easily accessed. The condition (3) is already implemented in commercial software packages such as COMSOL Multiphysics®. In recent years, solutions to similar problems utilizing open-source finite element software have been published^44,45,65^. However, they mostly focus on the time-domain formulation of (1). Thus, they do not utilise complex numbers, which are required to solve the problem described here. We used COMSOL Multiphysics®, V5.3a to solve (1). All computations were performed on a workstation with 24 physical Intel® Xeon® CPU Gold 6136, ${3.00}\,\hbox {GHz}$ cores and ${256}\,\hbox {GB}$ RAM.

A first numerical study on the effect of electrical stimulation on the membrane has been presented by Taghian et al.^18^ using a 2D domain of ${50}\,\upmu \hbox {m}$ height and ${100}\,\upmu \hbox {m}$ width with an abstract cell model (Fig. 2). The capacitive coupling in this model can be understood in terms of an equivalent circuit model. Two capacitors (${1}\,\upmu \hbox {m}$ thick insulation covering the electrodes) are connected in series with a parallel RC circuit (${48}\,\upmu \hbox {m}$ thick cell culture medium filling the space between the electrodes)^18^. We used the analytical formula for the impedance of a cylindrical, (lossy) dielectric

4 $$\begin{aligned} Z_i = \frac{d_i}{j \omega \varepsilon ^*_i \pi r_i^2} , \end{aligned}$$

to describe the total impedance of the circuit. Here, $\omega$ is the angular frequency, $d_i$ is the thickness of the cylinder, $r_i$ its radius and $\varepsilon ^*_i$ its complex permittivity. The complex permittivity, $\varepsilon ^*_i = \varepsilon _i - j \sigma _i / \omega$, contains the permittivity $\varepsilon _i$ and the conductivity $\sigma _i$. The electric field in the cell culture medium, which has shown to be influential on the TMP^18^, can be estimated from the equivalent circuit by taking the ratio of the cell culture medium impedance and the total impedance multiplied by the imposed voltage difference between the two electrodes. The numerical value of the average electric field can be computed by integrating the field over the volume of the cell culture medium and dividing the integrated value by the culture medium volume. All dielectric properties of the benchmark model are summarised in Table 1.

Recently, the aforementioned cell model was described in an axisymmetric setting^27^. This is based on the assumption of a cylindrical Petri dish, which is a valid choice in many cases. Moreover, the axisymmetric 2.5D approach mimics true 3D-behavior better than the 2D model presented in^18^, as the (hemi-)spherical shape of the cell is accounted for. In contrast, a pure 2D approach would assume the cell to be an infinitely long (hemi-)cylinder. Thus, we focused on the 2.5D model and performed all our 2D calculations under the assumption of axisymmetry.

In the existing models of capacitive-coupling set-ups, the cell membrane had a thickness of ${5}\,\hbox {nm}$ and was meshed explicitly^18,27^. We also meshed the membrane (Figs. S3 and S4) for a comparison to the case where the membrane is not discretised but described by (3). Due to the aspect ratio between the scales, the meshing is a numerically expensive and error-prone task^43^ if not performed with great care. To obtain an accurate result, we discretised the membrane such that it is represented by at least four layers of triangular elements. The mesh was additionally refined at the triple point where membrane, medium and insulator meet. This yielded 1,390,530 DOFs with quadratic Lagrange elements. Note that such a discretization appeared to be infeasible for the 3D case on our workstation. When making use of (3), the distance of the nodes on the membrane was set to be less than ${0.1}\,\upmu \hbox {m}$. In addition, the edges where the cell is in contact with the substrate were refined such that the results converged well. This yielded 31,713 DOFs. A 3D representation of the same system required 4,575,090 DOFs. Moreover, realistic 3D models of semi-ellipsoidal cells were studied (Fig. 3). These models mimic adherent cells^28^. For a set-up with one cell, 3,576,601 DOFs were required and for one with two cells 6,393,262 DOFs. The solution was evaluated along the membrane as shown in Figs. 2 and 3.

**Table 1:** Dielectric parameters for the numerical benchmark model as reported in^18,27^.

Domain	Quantity	Value
Insulator	Conductivity	${0}\,\hbox {S}/\hbox {m}$
	Relative permittivity	2.6
Culture medium	Conductivity	${1.5}\,\hbox {S}/\hbox {m}$
	Relative permittivity	80
Cytoplasm	Conductivity	${1.5}\,\hbox {S}/\hbox {m}$
	Relative permittivity	80
Cell membrane	Conductivity	${0}\,\hbox {S}/\hbox {m}$
	Relative permittivity	11.3

UQ becomes important when different model parameters are prone to substantial uncertainties. Since the parameters of biological systems are often not well known, we chose a mathematically rigorous way to address their influence on the model outcome. Each model parameter can be described by a probability distribution that represents the a priori knowledge of this parameter. The distribution can either be derived from experimental uncertainties, or based on prior expectations of the parameter value when experimental data is not available^70^. In the UQ approach, the forward problem is repeatedly solved for different parameter combinations corresponding to the assumed probability distributions of the individual parameters. To account for the uncertainty, i.e. the individual probability distributions, Monte Carlo (MC) sampling methods^71^ or the approximate but very efficient polynomial chaos (PC) methods^72^ can be employed. The efficiency of the PC methods stems from the fact that a polynomial expansion is used as a surrogate model. The polynomials are chosen with respect to the assumed probability distributions. In the next step, the polynomial expansion coefficients have to be found to construct the surrogate model. The equality of the model result and the polynomials, i.e. the surrogate model, is enforced at the collocation points. Recently, the open-source Python package *Uncertainpy* including both methods was published^40^. *Uncertainpy* generates parameter sets for which the model is evaluated and subsequently computes the polynomial expansion coefficients. The resulting surrogate model is then sampled to obtain the 5th and 95th percentile. A modified version of this package (https://github.com/j-zimmermann/uncertainpy/tree/1.2.0.1) was used for a PC analysis. We used the default settings of fourth-order polynomials (as recommended^73^), the point collocation method and 10,000 MC samples to compute the 5th and 95th percentile. In total, the PC approach for four uncertain parameters required 142 FEM model evaluations. For five uncertain parameters, the number of required model evaluations increased to 254. Obviously, it is advisable to reduce the number of uncertain parameters to a minimum. Note that MC methods would require considerably more evaluations (e.g. starting from 30,000 evaluations for four uncertain parameters^40^).

As a first UQ approach, we chose probability distributions relying on sensible assumptions based on our prior knowledge. These assumptions are summarised in Table 2. Since the dielectric properties of the cell culture medium can be measured with high accuracy^74^ and thus do not carry a large uncertainty, we kept them fixed in our analysis. Moreover, the dielectric properties of the insulating coating can be measured well, but the permittivity might change depending on the thickness of the coating^75^. Hence, we included the permittivity of the coating in the UQ analysis while assuming its conductivity to be negligibly small. For the measurement of the cellular parameters, only less accurate methods such as electrorotation, patch clamp or impedance spectroscopy are available^76^. Some of the parameters have also been covered in a previous study by Taghian et al.^18^. In this study, we intend to highlight the influence of the membrane conductivity; an aspect that has not been studied before. We probed conductivities from ${0}\,\hbox {S}/\hbox {m}$ (idealised case) to ${10^{-3}}\,\hbox {S}/\hbox {m}$ (extreme case, probably perforated membrane).

Experimental values for the dielectric parameters of chondrocytes, which we considered here, are available^37^. We used the reported values and errors for membrane conductivity, membrane permittivity, and cytoplasm conductivity of the PC5 cell line together with the reported average cell radius of ${4.1}\,\upmu \hbox {m}$ to study the effect of capacitive coupling for the set-up reported in^18^. Furthermore, we applied a membrane thickness of ${7}\,\hbox {nm}$ to be consistent with^37^. The assumed probability distributions are summarised in Table 3. Note that the relatively large conductivity is supported by other experimental findings reporting a large permeability of the chondrocyte membrane at rest for certain ions^77,78^. Eventually, we propagate the uncertainties of the cellular parameters through the model using the PC UQ technique.

The UQ analysis yields the mean value, variance, and the $90\%$ prediction interval of the parameter under investigation. Moreover, the first-order and total Sobol indices for each uncertain parameter are computed. In general, Sobol indices serve for a variance-based sensitivity analysis. The first-order Sobol indices reveal the individual influence of each parameter on the variance of the TMP value. The maximal possible value for the sum of first-order Sobol indices is one^79^. The total Sobol indices cover both influences and their sum can exceed one.

**Table 2:** Parameters for the UQ study of the numerical model as reported in^18,27^. ${{\mathscr {U}}}$ stands for uniform distribution.

Domain	Quantity	Value	explanation
Cytoplasm	Conductivity	${{\mathscr {U}}}(1,1.5) [\hbox {S}/\hbox {m}]$	Guess
	Relative permittivity	${{\mathscr {U}}}(60, 80)$	Assumptions from^37^ and^18^
Cell membrane	Conductivity	${{\mathscr {U}}}(0, {10^{-5}}) [\hbox {S}/\hbox {m}]$	Possible range
	Relative permittivity	${{\mathscr {U}}}(9.9, 12.1)$	$10\%$ variation
Coating	Conductivity	0﻿ S/m	
	Relative permittivity	${{\mathscr {U}}}(2.4, 2.8)$	Guess based on^75^

**Table 3:** Parameters for the UQ study of the numerical model as reported in^18,27^ applied on chondrocytes (values based on^37^). ${{\mathscr {N}}}$ stands for normal distribution. Note that the cytoplasm permittivity was kept fixed in the analysis in^37^.

Domain	Quantity	Value
Cytoplasm	Conductivity	${{\mathscr {N}}}(0.12, 0.02) [\hbox {S}/\hbox {m}]$
	Relative permittivity	60
Cell membrane	Conductivity	${{\mathscr {N}}}({6.895 \times 10^{-5}}, {1.77 \times 10^{-5}}) [\hbox {S}/\hbox {m}]$
	Relative permittivity	${{\mathscr {N}}}(59.06, 12.88)$
Coating	Conductivity	0﻿ S/m
	Relative permittivity	${{\mathscr {U}}}(2.4, 2.8)$

## Supplementary Information

Supplementary Information.
