# Supplementary material for: Numerical study on the effect of capacitively coupled electrical stimulation on biological cells considering model uncertainties
Source: Sci Rep. 2022 Mar 18;12:4744. doi: 10.1038/s41598-022-08279-w (PMC8933463; doi:10.1038/s41598-022-08279-w)
Supplement: Supplementary file 1 — Supplementary Information. [file 41598_2022_8279_MOESM1_ESM.pdf]

Electronic Supplementary Material: Numerical study on the effect of capacitively coupled electrical stimulation on biological cells considering model uncertainties

Julius Zimmermann[1,*], Richard Altenkirch[2], and Ursula van Rienen[1,3,4,*]

[1]Institute of General Electrical Engineering, University of Rostock, D-18051 Rostock, Germany
[2]Institute of Physics, University of Rostock, D-18051 Rostock, Germany
[3]Department Life, Light & Matter, University of Rostock, D-18051 Rostock, Germany
[4]Department of Ageing of Individuals and Society, Interdisciplinary Faculty, University of Rostock, D-18051 Rostock, Germany
[*]julius.zimmermann@uni-rostock.de, ursula.van-rienen@uni-rostock.de

Electroquasistatic approach and choice of material properties

As no experimental data were available for the dielectric properties of the cell culture medium used in our study, we searched for experimental data for a similar system. The temperature and frequency dependence of the conductivity have been discussed elsewhere[1]. These sources indicate that no significant frequency dependence has been observed between 50 Hz and 1 kHz. A study including higher frequencies has been carried out for sodium chloride[2]. Sodium chloride is the main component of many cell culture media and can thus be used for comparison. Fig. S1 shows the dielectric properties of a sodium chloride solution with a conductivity that resembles the usually assumed values for cell culture media. The values are based on the experimental data presented in a previous work[2].

The electroquasistatic (EQS) approach is justified when the electromagnetic wavelength is much longer than the characteristic length d of the system[3]. This can be expressed by the inequality

$$|kd| \ll 1 \quad , \tag{1}$$

where k is the wave number, which is inversely proportional to the wavelength, and reads

$$k = \omega\sqrt{\mu\varepsilon\left(1 - \frac{j\sigma}{\omega\varepsilon}\right)} \quad . \tag{2}$$

For heterogeneous systems as studied here, the values for permittivity ε as well as conductivity σ should be the maximal occurring values[4]. The different limits of (1) are shown in Fig. S2. The dielectric properties given in Table 1 in the main text were assumed.

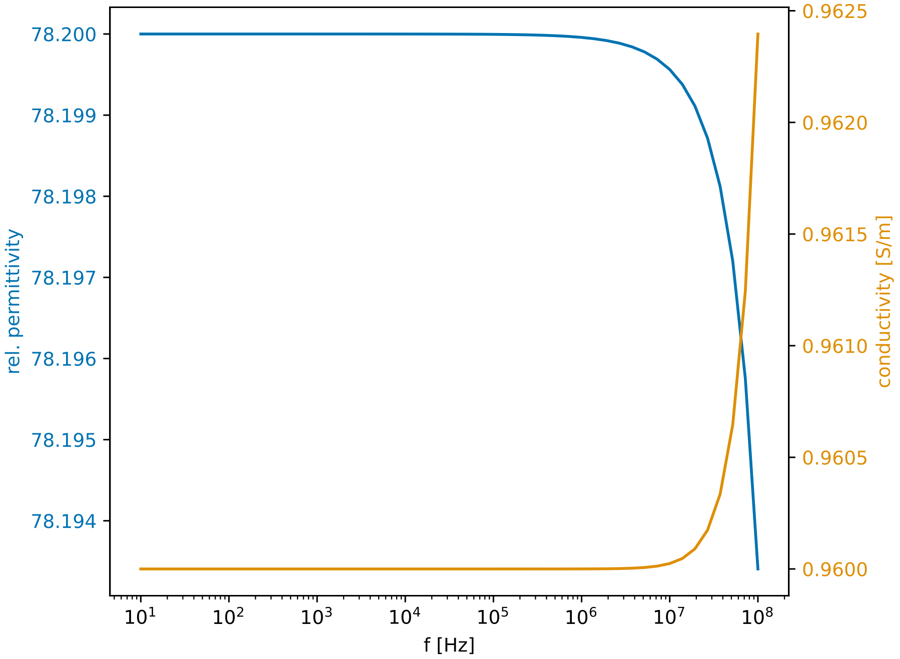

Figure S1. Evaluation of the dielectric properties of NaCl solution with (expectedly) similar properties to the actual cell culture medium for the relevant frequency range. The properties of the NaCl solution were computed using the Cole-Cole model and experimental results that were reported in a previous work[2].

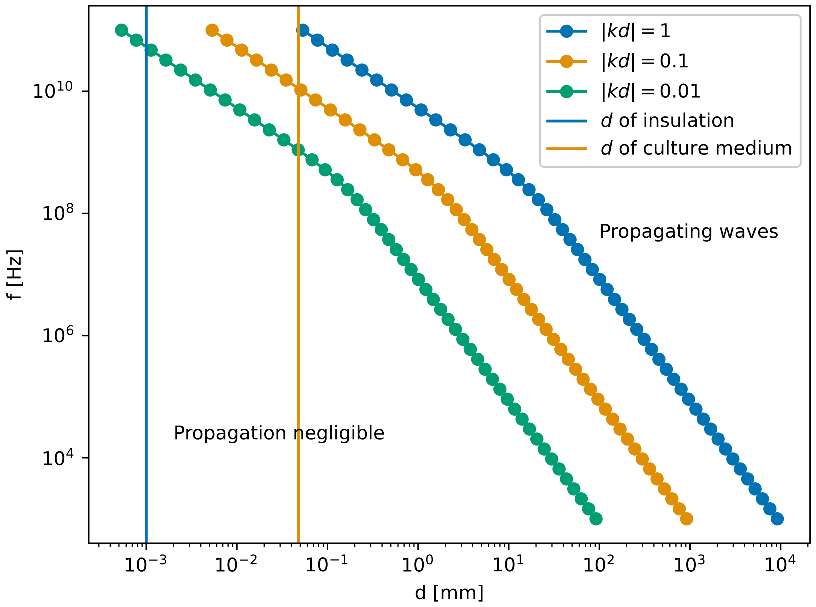

Figure S2. Evaluation of the characteristic size d and the frequency values, from which on the EQS formulation is no longer applicable. The wavenumber k is used as formulated previously[3]. The inequality $|kd| \ll 1$, which needs to be fulfilled to permit the use of EQS, is visualised for different $|kd|$. Furthermore, the characteristic sizes of the insulating layer and the cell culture medium are shown to highlight the frequencies from which on EQS should not be used.

Details of the numerical model

When the membrane was meshed explicitly, we discretized the membrane such that it is represented by at least four layers of triangular elements and even finer at the triple point, where the membrane has a rather sharp edge (Fig. S3).

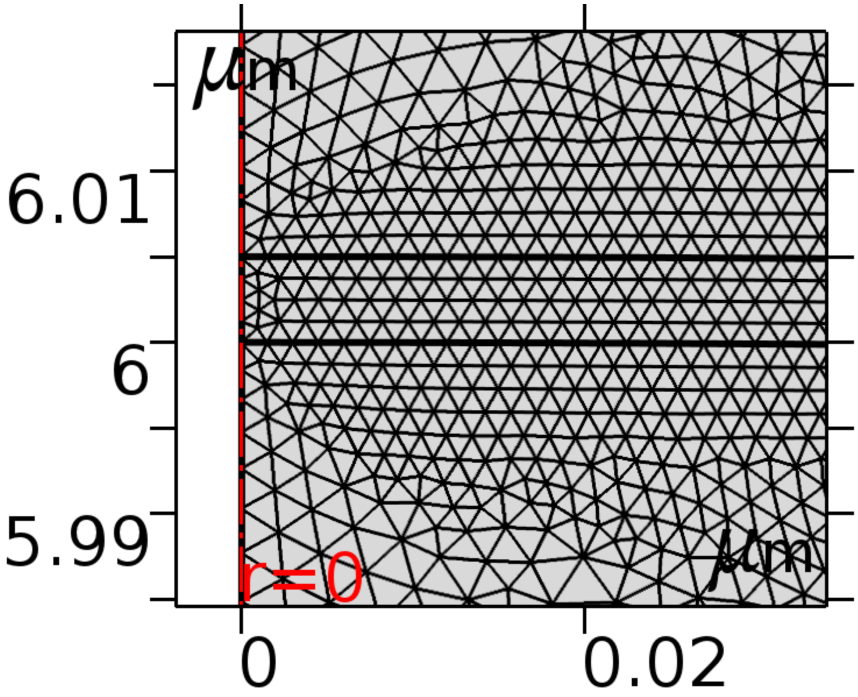

Figure S3. Discretization of the cell membrane (between 6 and 6.005 µm) at the cell's apex. The symmetry axis is shown in red.

A critical point in the evaluation was located close to the triple point (Fig. S4). The same figure shows an example of how the TMP was evaluated.

In Fig. S5, we show exemplary points at which the TMP was evaluated.

Validation of the numerical approach

A first numerical study on the effect of electrical stimulation on the membrane has been presented by Taghian *et al.*[5] using a 2D domain of 50 µm height and 100 µm width with an abstract cell model (Fig. S6b).

The capacitive coupling in this model can be understood in terms of an equivalent circuit model. Two capacitors (1 µm thick insulation covering the electrodes) are connected in series with a parallel RC circuit (48 µm thick cell culture medium filling the space between the electrodes)[5]. We used the analytical formula for the impedance of a cylindrical, (lossy) dielectric

$$Z_i = \frac{d_i}{j\omega\varepsilon_i^* \pi r_i^2} \quad , \tag{3}$$

to describe the total impedance of the circuit. Here, ω is the angular frequency, d_i is the thickness of the cylinder, r_i its radius and ε_i^* its complex permittivity. The complex permittivity, $\varepsilon_i^* = \varepsilon_i - j\sigma_i/\omega$, contains the permittivity ε_i and the conductivity σ_i. The electric field in the cell culture medium, which has shown to be influential on the TMP[5], can be estimated by taking the ratio of the cell culture medium impedance and the total impedance multiplied by the imposed voltage difference between the two electrodes. All dielectric properties of the benchmark model are summarised in Table 1 of the manuscript. The deviation between the analytical and the numerical solution is negligibly small (Fig. S7). However, the relative error grows with decreasing frequency below 100 Hz.

To validate the cell model, the approximate method is compared against the full-fidelity model at prominent points along the cell membrane (Figs. S4 and S6). The TMP in the electro-quasistatic formulation is a phasor. Thus, its absolute value and phase are computed to check the validity of the approximate method in comparison to the so-far employed full-fidelity method. Apart from this comparison, we generally report the absolute value of the TMP as this is the property of interest in therapeutic applications.

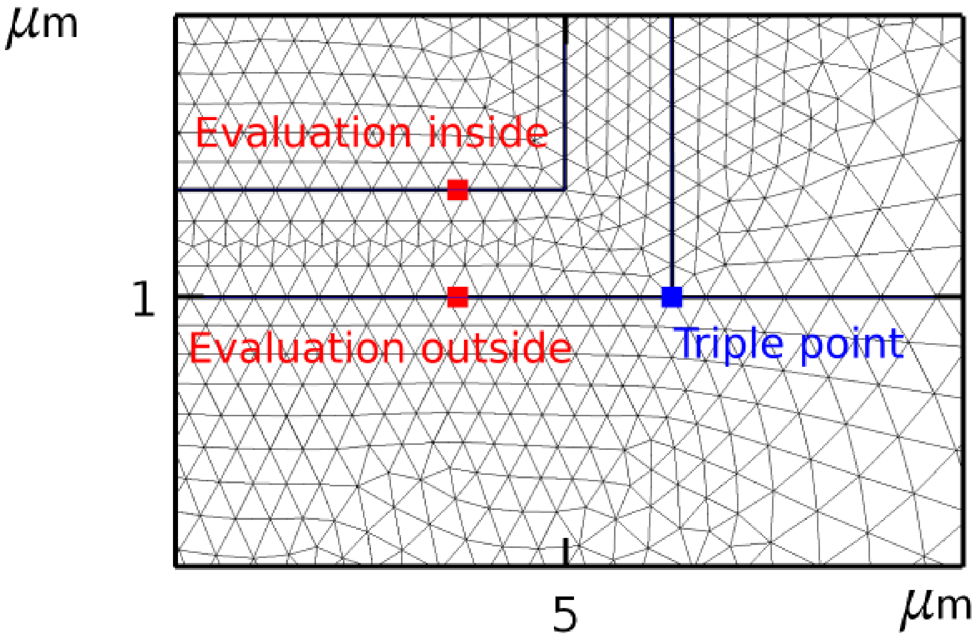

Figure S4. Discretization of the cell membrane at the cell's right corner close to the triple point. The evaluation along the membrane is presented for one of the evaluation points shown in Fig. S5.

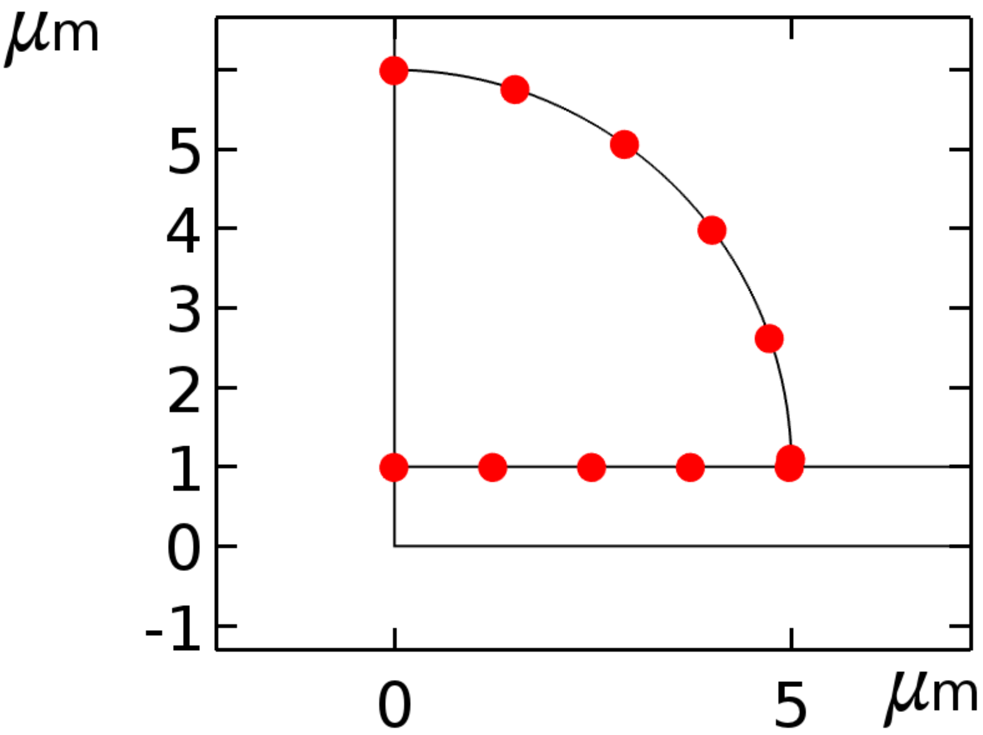

Figure S5. Points for evaluation of TMP. Note that in this illustration, the triple point is covered by two points, one on the circular and one on the bottom line part of the cell membrane.

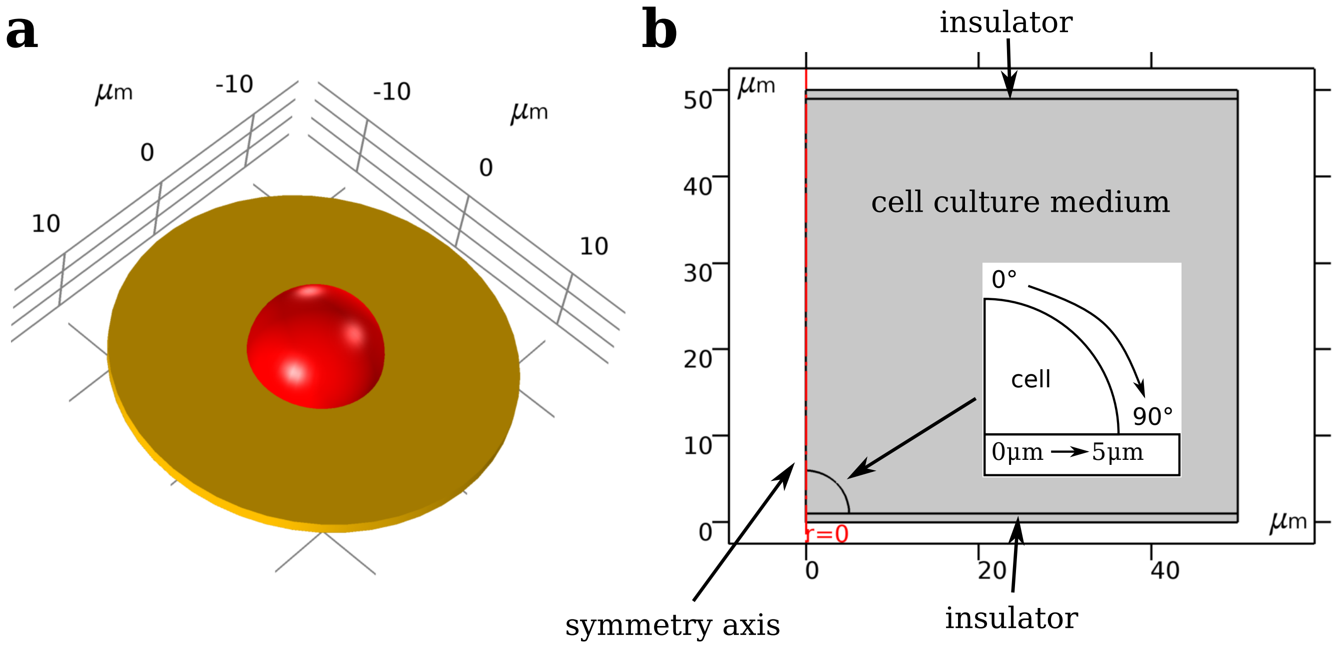

Figure S6. 2.5D model of a cell on a substrate exposed to capacitively-coupled fields[5]. The 3D equivalent of the 2.5D model (zoomed-in) is shown in (**a**). The cell (red) adheres to a substrate (yellow) which is a plastic insulator with a thickness of 1 µm. The cell has a radius of 5 µm and its membrane a thickness of 5 nm. The 2D view of the 2.5D model is shown in (**b**). On the top and bottom boundaries of the domain, Dirichlet boundary conditions are applied to impose a net voltage difference. The boundary conditions mimic the electrodes, which are not explicitly modelled. Note that the electrodes are not in direct contact with the medium since they are covered by insulators. The other boundaries are electrically insulating. Material parameters for the cell cytoplasm and the culture medium are assigned as stated in Table 1 in the manuscript. Different locations along the curved part of the cell membrane are denoted by the angle with the symmetry axis. Positions along the bottom part are denoted by the distance to the cell centre.

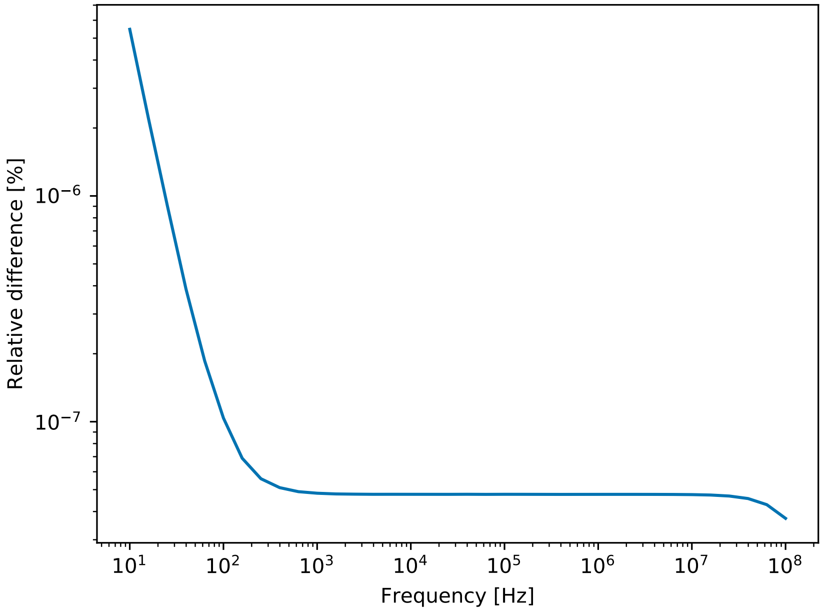

Figure S7. Relative difference between the analytically determined and the numerical FEM result of the electric field strength in the cell culture medium for the benchmark system considered in previous works[5,6]. Due to the geometry and the boundary conditions, the field is homogeneous in the cell culture medium. The numerical result corresponds to the average field in the medium.

Firstly, the TMP was computed for the same dielectric parameters as in previous works[5,6] (see Table 1 in manuscript) using the full-fidelity as well as the approximate model. The absolute value of the TMP along the circular part of the membrane is shown in Fig. S8. Up to about 1 MHz it remains constant and does not change along the membrane. From then on it starts to change, depending on the point on the membrane. Figure S8 shows that at the membrane apex (denoted by the blue line, i.e. an angle of 0°), the TMP increases from about 1 MHz and peaks at about 10 MHz before it decreases. A special point on the membrane is the triple point, where membrane, medium and insulator meet (see also Fig. S4). On the circular part, the triple point is located at an angle of 90°. Close to this point, the TMP drops continuously from about 1 MHz on and does not peak. The approximate and the full-fidelity method do not deviate significantly. For frequencies up to 100 kHz, the TMP along the bottom line (Fig. S10) is roughly 1.9 times larger than that along the circular part and is constant except for the triple point.

To assess the accuracy of the approximate method in a straightforward manner, we compared the relative error of the result at different points along the membrane with the results of the full-fidelity model as the best possible approximation. Corresponding figures can be found in Figs. S12– S15). For all points except the triple points, the relative error of the TMP remains below 0.1%. On the bottom line, the relative error is actually only about 10^{-5}% for most of the frequencies. Close to the triple point, the difference increases and reaches more than 1% on the circular part and more than 0.1% on the bottom line for high frequencies close to 100 MHz, respectively. In contrast, the phase values are more sensitive to the computational method. For small frequencies up to 1 kHz, the results deviate even more than 100%. Note that the phase in this frequency region is close to 0°, and the absolute difference is thus only a few degrees (less than 4° for both the circular part and the bottom line). For larger frequencies, the relative error drops again below 1%.

Parameter dependence: membrane conductivity

The TMP along the bottom line for different conductivities is shown in Fig S16. In addition, its phase is shown in Fig. S17.

Uncertainty Quantification

The results of the UQ analysis of the basic model at the cell bottom are shown in Fig. S18. When using the experimentally determined dielectric properties of chondrocytes, the analysis provides different results for the cell bottom (Fig. S19).

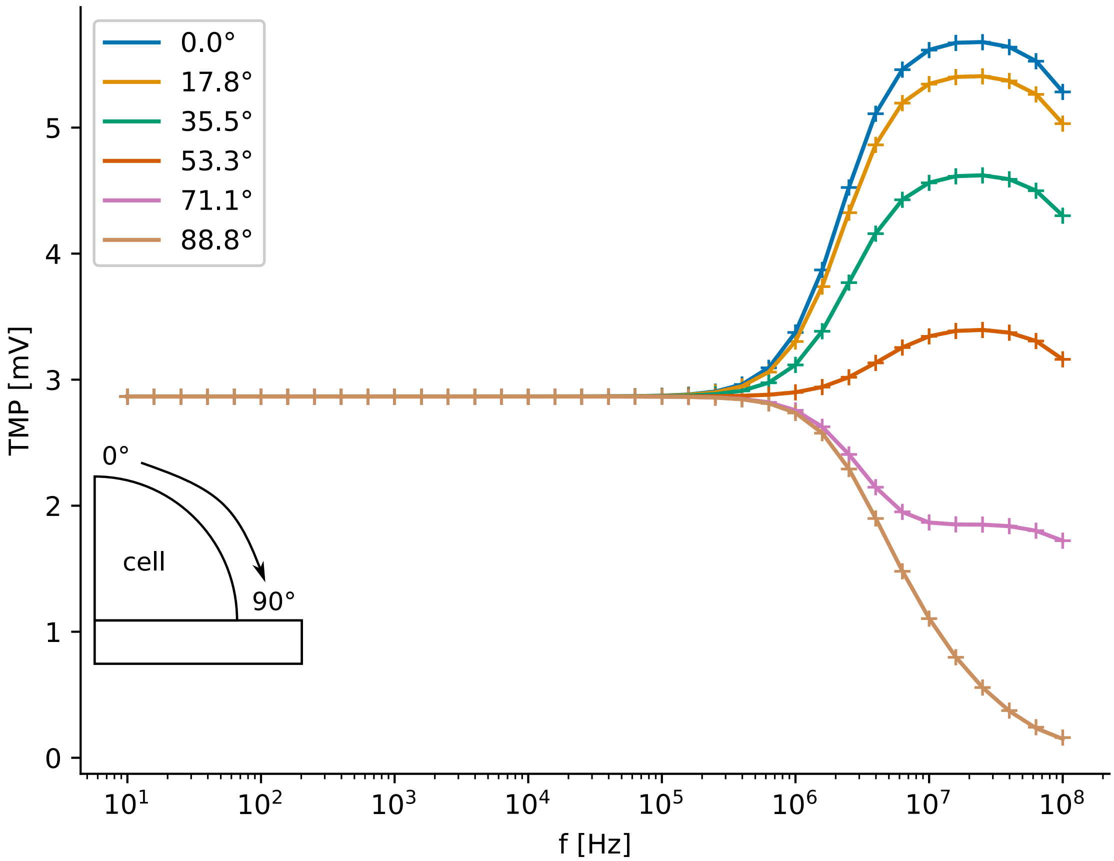

Figure S8. TMP for a membrane conductivity of $0\,\mathrm{S\,m^{-1}}$ for the 2.5D model along the circular part. The full-fidelity model (solid lines) is compared to the approximate solution at different points on the membrane. The points are characterised by the angle between field and vector from the centre of the cell at $(0\,\mu\mathrm{m}, 1\,\mu\mathrm{m})$, i.e. the blue curve corresponds to the cell apex.

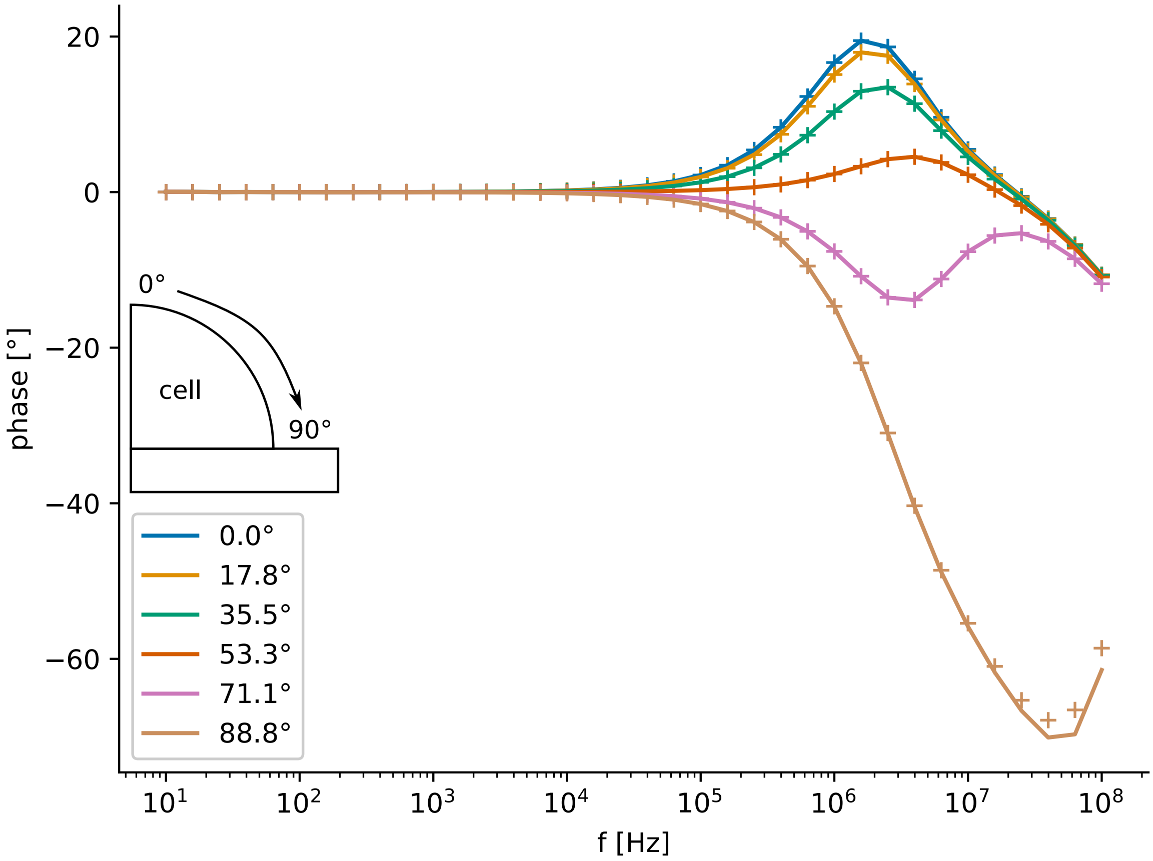

Figure S9. Phase of the TMP for a membrane conductivity of $0\,\mathrm{S\,m^{-1}}$ for the 2.5D model along the circular part. The full-fidelity model (solid lines) is compared to the approximate solution at different points on the membrane. The points are characterised by the angle between field and vector from the centre of the cell at $(0\,\mu\mathrm{m}, 1\,\mu\mathrm{m})$, i.e. the blue curve corresponds to the cell apex.

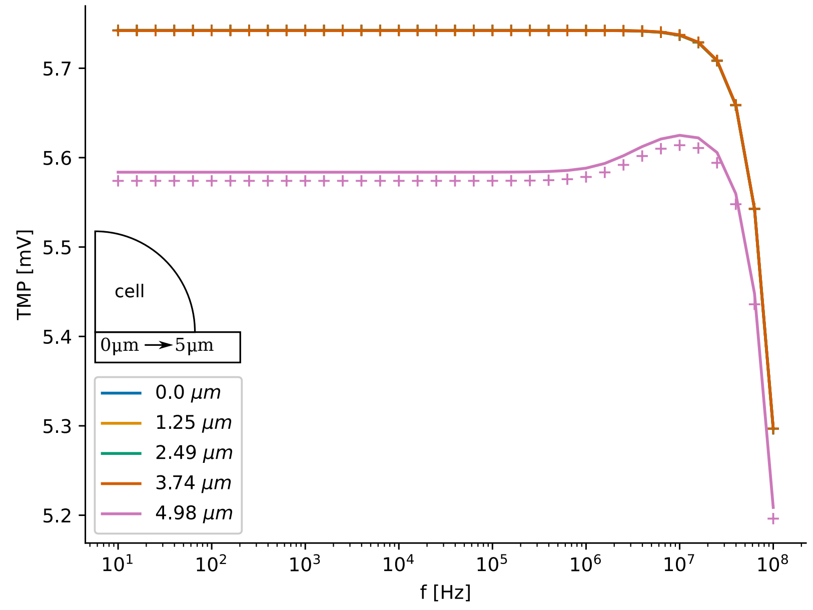

Figure S10. TMP for a membrane conductivity of $0\,\mathrm{S\,m^{-1}}$ for the 2.5D model along the bottom line. The full-fidelity model (solid lines) is compared to the approximate solution at different points on the membrane. The points are characterized by the distance to the center at $x = 0\,\mu\mathrm{m}$, i.e. all lines except for the point with a distance of $4.98\,\mu\mathrm{m}$ overlap.

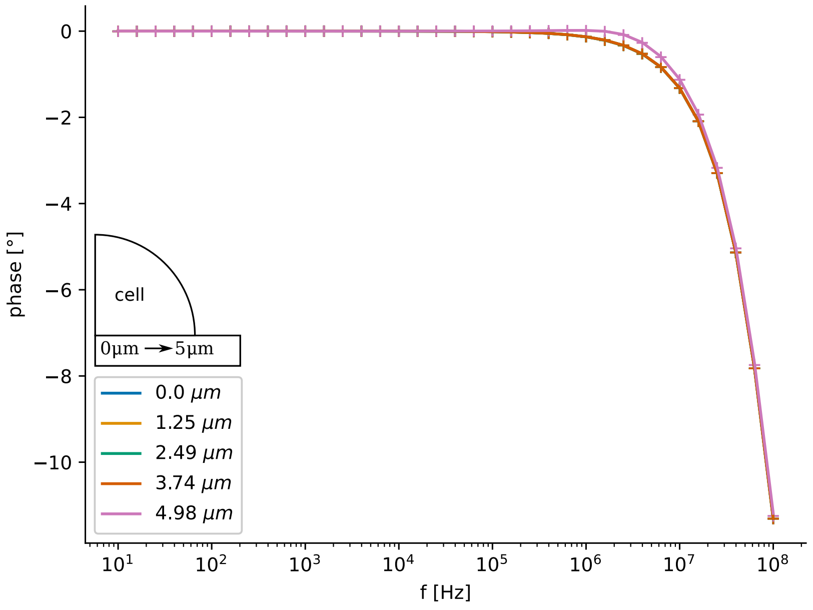

Figure S11. Phase of TMP for a membrane conductivity of $0\,\mathrm{S\,m^{-1}}$ for the 2.5D model along the bottom line. The full-fidelity model (solid lines) is compared to the approximate solution at different points on the membrane. The points are characterized by the distance to the center at $x = 0\,\mu\mathrm{m}$, i.e. all lines except for the point with a distance of $4.98\,\mu\mathrm{m}$ overlap.

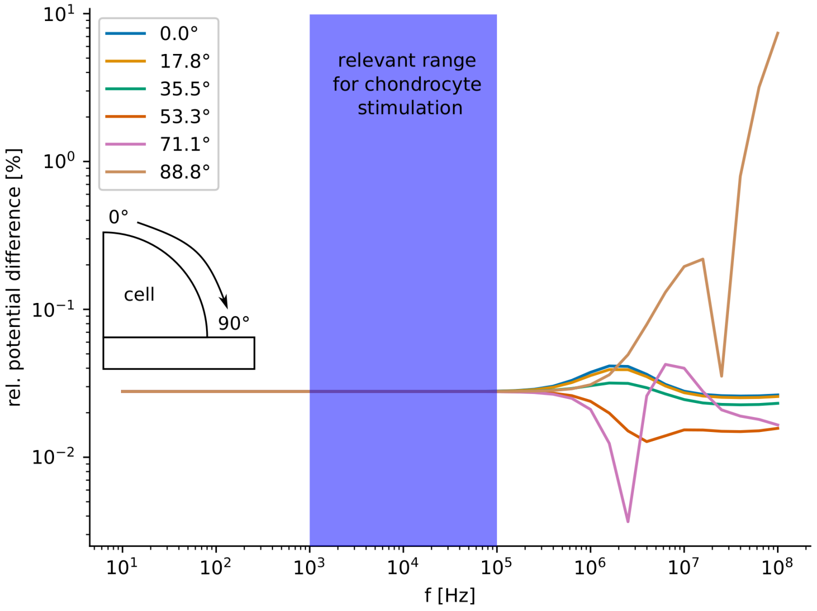

Figure S12. Relative error of the TMP along the circular part in case of a membrane conductivity of $0\,\mathrm{S\,m^{-1}}$ for the 2.5D model in comparison with the full-fidelity model. The corresponding TMP is shown in the main manuscript (Fig. 6).

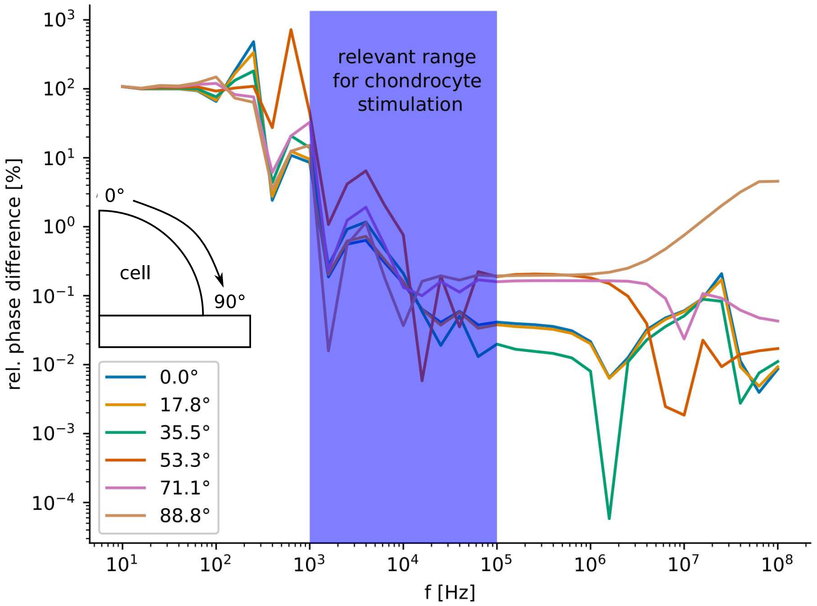

Figure S13. Relative error of the phase of the TMP along the circular part in case of a membrane conductivity of $0\,\mathrm{S\,m^{-1}}$ for the 2.5D model in comparison with the full-fidelity model. The corresponding TMP is shown in the main manuscript (Fig. 7).

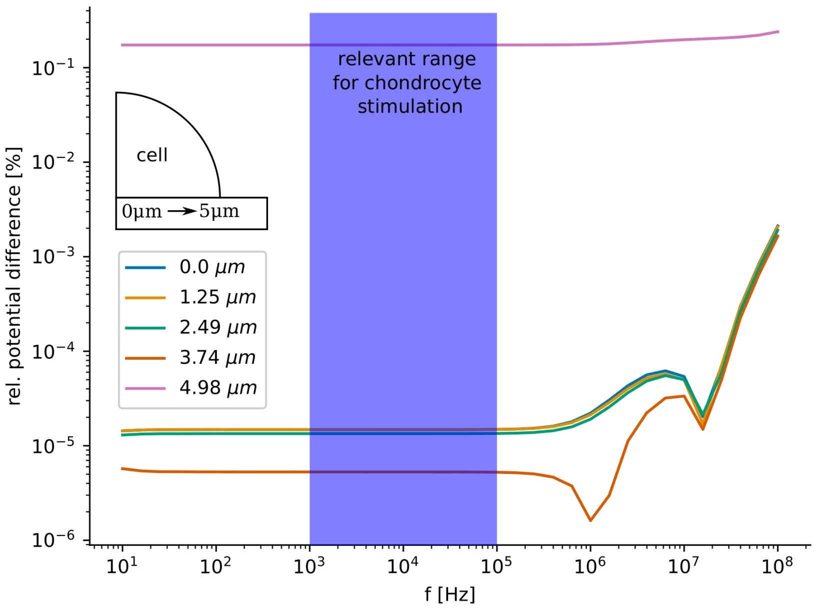

Figure S14. Relative error of the TMP along the bottom line in case of a membrane conductivity of $0\,\mathrm{S\,m^{-1}}$ for the 2.5D model in comparison with the full-fidelity model as shown in Fig. 10.

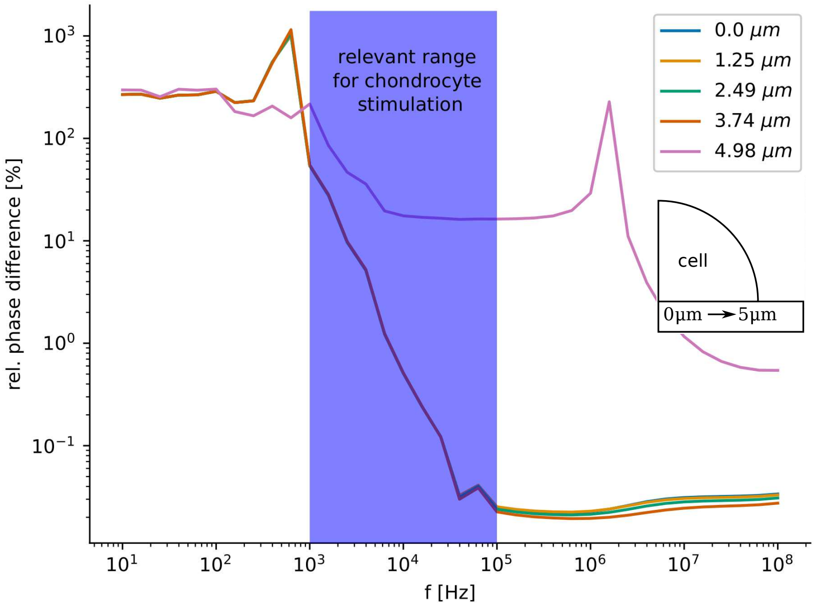

Figure S15. Relative error of the phase of the TMP along the bottom line in case of a membrane conductivity of $0\,\mathrm{S\,m^{-1}}$ for the 2.5D model in comparison with the full-fidelity model.

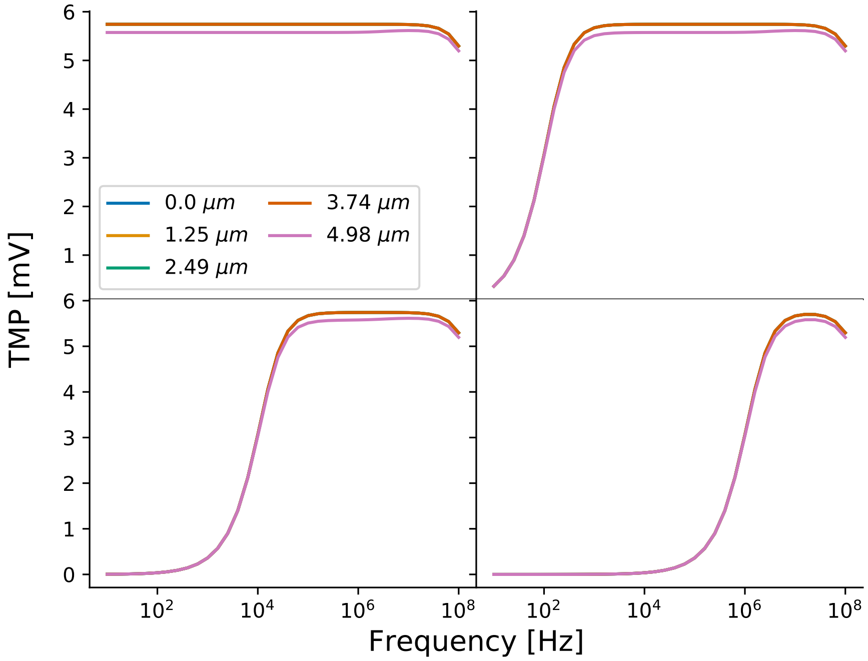

Figure S16. TMP along the bottom line of the cell membrane for different conductivities of $0\,\mathrm{S\,m^{-1}}$ (upper left), $1\times10^{-7}\,\mathrm{S\,m^{-1}}$ (upper right), $1\times10^{-5}\,\mathrm{S\,m^{-1}}$ (bottom left) and $1\times10^{-3}\,\mathrm{S\,m^{-1}}$ (bottom right). The results were generated using the approximate method.

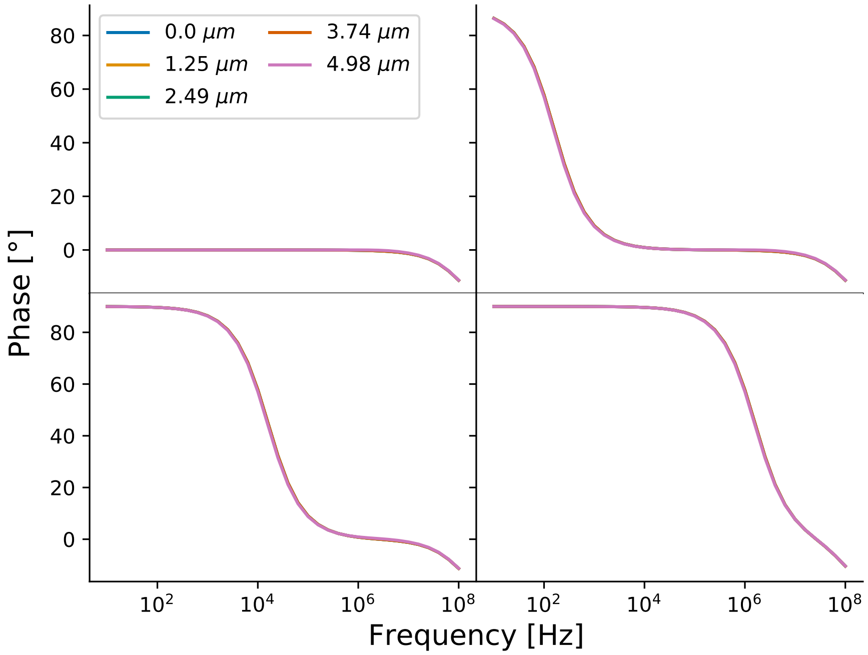

Figure S17. Phase along the bottom line of the cell membrane for different conductivities of $0\,\mathrm{S\,m^{-1}}$ (upper left), $1\times10^{-7}\,\mathrm{S\,m^{-1}}$ (upper right), $1\times10^{-5}\,\mathrm{S\,m^{-1}}$ (bottom left) and $1\times10^{-3}\,\mathrm{S\,m^{-1}}$ (bottom right). The results were generated using the approximate method.

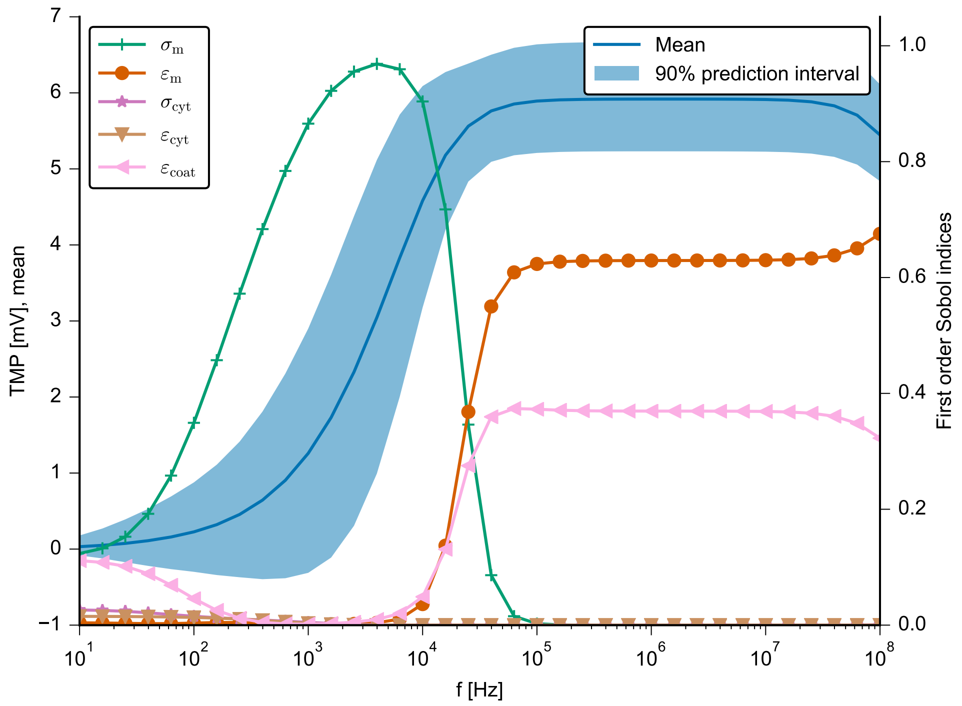

Figure S18. Left axis: Mean and 90% prediction interval of the absolute value of the TMP at the cell bottom for the basic benchmark model. Right axis: First order Sobol indices for each uncertain parameter, i.e. the conductivity (dark green) and permittivity (orange) of the membrane, the conductivity (purple) and permittivity (brown) of the cytoplasm, and the permittivity of the coating (pink), respectively.

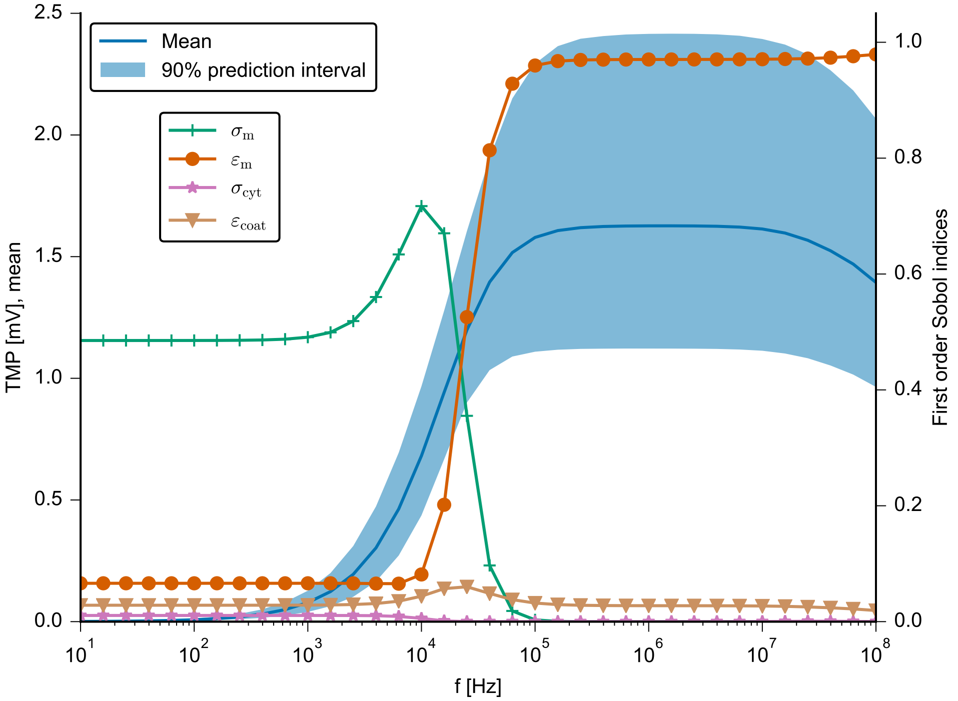

Figure S19. Left axis: Mean and 90% prediction interval of the absolute value of the TMP at the cell bottom for the basic chondrocyte model. Right axis: First order Sobol indices for each uncertain parameter, i.e. the conductivity (dark green) and permittivity (orange) of the membrane, the conductivity (pink) of the cytoplasm, and the permittivity of the coating (brown), respectively

References

1. Mazzoleni, A. P., Sisken, B. F. & Kahler, R. L. Conductivity values of tissue culture medium from 20°C to 40°C. *Bioelectromagnetics* **7**, 95–99, DOI: 10.1002/bem.2250070111 (1986).
2. Peyman, A., Gabriel, C. & Grant, E. H. Complex permittivity of sodium chloride solutions at microwave frequencies. *Bioelectromagnetics* **28**, 264–274, DOI: 10.1002/bem.20271 (2007).
3. van Rienen, U. *et al.* Electro-quasistatic simulations in bio-systems engineering and medical engineering. *Adv. Radio Sci.* **3**, 39–49 (2005).
4. Dirks, H. K. Quasi-stationary fields for microelectronic applications. *Electr. Eng.* **79**, 145–155, DOI: 10.1007/BF01232924 (1996).
5. Taghian, T., Narmoneva, D. A. & Kogan, A. B. Modulation of cell function by electric field: a high-resolution analysis. *J. R. Soc. Interface* **12**, 20150153–20150153 (2015).
6. Escobar, J. F., Vaca-González, J. J. & Garzón-Alvarado, D. A. Effect of magnetic and electric fields on plasma membrane of single cells : A computational approach. *Eng. Reports* **e12125**, 1–14, DOI: 10.1002/eng2.12125 (2020).
